# Stimuli-responsive oligonucleotides in prodrug-based approaches for gene silencing

**DOI:** 10.3762/bjoc.14.32

**Published:** 2018-02-19

**Authors:** Françoise Debart, Christelle Dupouy, Jean-Jacques Vasseur

**Affiliations:** 1 IBMM, Université de Montpellier, CNRS, ENSCM, Montpellier, France

**Keywords:** enzymolabile group, light-responsive group, oligonucleotide prodrugs, reduction-responsive, stimuli-responsive nucleic acids, thermolytic prodrugs

## Abstract

Oligonucleotides (ONs) have been envisaged for therapeutic applications for more than thirty years. However, their broad use requires overcoming several hurdles such as instability in biological fluids, low cell penetration, limited tissue distribution, and off-target effects. With this aim, many chemical modifications have been introduced into ONs definitively as a means of modifying and better improving their properties as gene silencing agents and some of them have been successful. Moreover, in the search for an alternative way to make efficient ON-based drugs, the general concept of prodrugs was applied to the oligonucleotide field. A prodrug is defined as a compound that undergoes transformations in vivo to yield the parent active drug under different stimuli. The interest in stimuli-responsive ONs for gene silencing functions has been notable in recent years. The ON prodrug strategies usually help to overcome limitations of natural ONs due to their low metabolic stability and poor delivery. Nevertheless, compared to permanent ON modifications, transient modifications in prodrugs offer the opportunity to regulate ON activity as a function of stimuli acting as switches. Generally, the ON prodrug is not active until it is triggered to release an unmodified ON. However, as it will be described in some examples, the opposite effect can be sought.

This review examines ON modifications in response to various stimuli. These stimuli may be internal or external to the cell, chemical (glutathione), biochemical (enzymes), or physical (heat, light). For each stimulus, the discussion has been separated into sections corresponding to the site of the modification in the nucleotide: the internucleosidic phosphate, the nucleobase, the sugar or the extremities of ONs. Moreover, the review provides a current and detailed account of stimuli-responsive ONs with the main goal of gene silencing. However, for some stimuli-responsive ONs reported in this review, no application for controlling gene expression has been shown, but a certain potential in this field could be demonstrated. Additionally, other applications in different domains have been mentioned to extend the interest in such molecules.

## Introduction

For past decades, oligonucleotide-based therapies have been widely developed using short synthetic oligonucleotides (ONs) and their chemically modified mimics as powerful tools to block mRNA function, inhibit protein function or induce an immune response [1–2]. Among these ON therapeutic strategies, ON-based gene silencing, which involves mRNAs as specific targets, has been largely investigated, and several promising ONs have been under clinical development [3]. Gene silencing strategies include antisense oligonucleotides (AONs), ribozymes, DNAzymes, small interfering RNAs (siRNAs) and micro RNAs (miRNAs) that specifically target the complementary mRNA sequence of the relevant undesired gene before translation.

AONs are single-stranded DNA of 15 to 25 nucleotides in length that bind to mRNA targets through Watson–Crick base pairing and form a RNA/DNA duplex [4]. This can result in either mRNA cleavage mediated by RNase H or mRNA translational arrest through steric blocking. Another strategy for gene inhibition involves ribozymes [5] and DNAzymes [6], which are nucleic acid molecules with enzymatic activity. These catalytic RNAs and DNAs trigger the cleavage of RNA substrates at a specific position. Additionally, ribozymes can catalyze the ligation of target mRNA, extending their therapeutic potential to RNA repair applications. Finally, another promising ON-based therapy, more potent than AONs or ribozymes for gene knockdown, is centered on the RNA interference (RNAi) mechanism, which uses two natural pathways for gene silencing. One is guided by double-stranded siRNAs of 19–23 nucleotides in length that are fully complementary to the mRNA targets, and the other is guided by miRNAs (22 nucleotides in length) that bind incorrectly within the 3’-untranslated region (3’-UTR) of the target mRNAs [7]. miRNAs also represent interesting targets, and inhibition of their function was obtained using anti-miRNA AONs via an antisense approach or via the blocking of the mRNA binding site (miRNA masking) [8].

Although many ONs are under investigation for clinical use, several hurdles remain to be overcome for the exploitation of ONs as therapeutic compounds. Among the major limitations of unmodified ONs, poor stability in vivo, low delivery and lack of specificity to target cells or tissues, off-target effects and toxicity hamper the path to success of ON-based therapeutics and need to be solved. Fortunately, various chemical modifications of ONs have been designed to address these issues [9]. The most common modification in AONs and siRNAs is the phosphorothioate (PS) backbone in the replacement of the phosphate ester internucleotide linkages. This modification provides nuclease stability and favorable pharmacokinetic properties but can lead to some toxicity. In addition, the most extensively used sugar modifications are represented by the 2’-modifications: 2’-*O*-methyl (2’-OMe), 2’-fluoro (2’-F), and 2’-*O*-(2-methoxyethyl) (MOE) [9–10]. Some examples of the combination of 2’-OMe and 2’-F modified nucleotides in siRNAs were reported, and the potency of the modified siRNA was increased compared to unmodified siRNA. Many chemical modifications have been introduced in ONs definitively as a means of modifying and better improving their properties as gene silencing agents [11]. However, an alternative way to make efficient ON-based drugs is to apply the general concept of prodrugs to the oligonucleotide field. Based on the definition of a prodrug given by Albert in 1958 [12], a prodrug is an agent that undergoes chemical or enzymatic transformations in vivo to yield the active parent drug. The prodrug approach is used to optimize the physicochemical properties of the drug and to improve its pharmacological and toxicological profile.

Oligonucleotide prodrugs that could be defined as caged oligonucleotides are transiently modified ONs with non-permanent chemical modifications (responsive units) that can be removed in response to appropriate stimuli, producing the native oligonucleotide. The aim of the prodrug strategy for nucleic acid therapeutic applications such as gene regulation is to circumvent the poor chemical stability of nucleic acids in biological media due to their low resistance to nucleases and to overcome their low cell uptake due to their polyanionic nature. In the present review, we aimed to identify various ON prodrugs that are responsive to various stimuli and evaluate their applications, mainly focusing on the control of gene expression. The use of ON prodrugs as aptamers, decoys or immunostimulatory ONs in other ON-based therapeutic strategies is marginally mentioned.

Two classes of stimuli can trigger inactive ON prodrugs in active biomolecules. Here, we summarize the chemically modified ONs that are responsive to either internal biochemical regulatory stimuli such as glutathione or enzymes (reductases, carboxyesterases), or external physical stimuli such as heat or light (photoirradiation). The transient responsive units may be attached at different positions of the ON: the internucleotide linkage, the ribose, the nucleobase, or the 5’ or 3’ extremities. For simplicity, each section corresponding to one class of stimulus has been divided into sub-sections related to the site of the modification in the ON when the subject was thoroughly documented.

## Review

### Reduction-responsive ONs

These modified ONs are responsive to the reducing environment inside cells due to the natural presence of glutathione (GSH) as a conversion trigger. ONs that are responsive to the action of reductases under hypoxic conditions will be discussed vide infra in a separate section. The intracellular concentration of GSH ranges from 1 mM to 10 mM, which is 10–100 times higher than its extracellular concentration. Consequently, ON prodrugs should be stable outside the cell and, after cellular uptake, would be converted into the native ONs by intracellular abundant GSH. In this context, two classes of reduction-responsive units, disulfide-bond and benzyl-containing groups, were mainly introduced in prodrug-based ONs.

#### Modifications at the internucleotide linkage

**Scheme 1:**
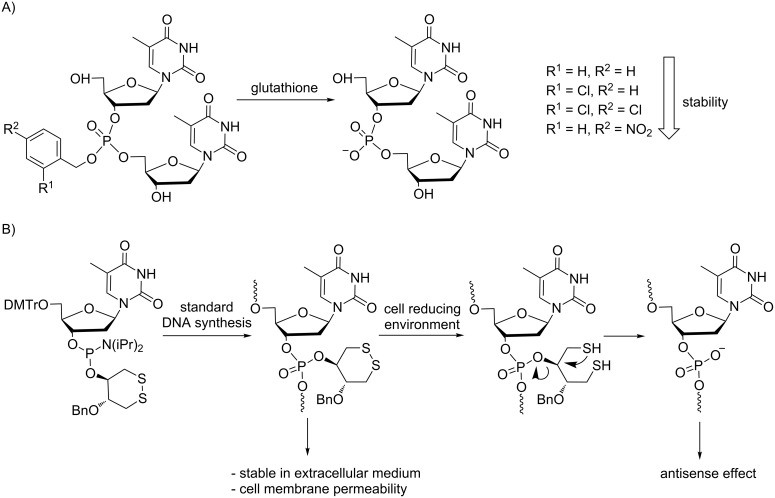
Demasking under reducing agents of ON prodrugs modified as phosphotriesters with A) benzyl groups [13] and B) a cyclic disulfide *trans*-5-benzyl-1,2-dithiane-4-yl moiety [14].

Masking the negative charges of native phosphates typically improves cell penetration of the modified ONs in addition to an increase in their nuclease resistance. Thus, two Japanese groups have proposed prodrug-type phosphotriester ONs responsive to GSH (Scheme 1) [13–14]. Ono presented a preliminary study on a model of a thymidine dimer with differently substituted benzyl groups at the internucleotide linkage [13]. It was shown that the stability in aqueous buffer and deprotection rates in the presence of GSH were influenced by the nature of substituents (Cl, NO_2_) on the benzene ring. More recently, Urata et al. reported a reduction-responsive modification containing a typical disulfide bond within a robust cyclic disulfide moiety [14]. Several modified ONs containing the cyclic disulfide *trans*-5-benzyl-1,2-dithiane-4-yl moiety have been synthesized using the corresponding thymidine phosphoramidite. Although they exhibited strong stability in serum and penetrated cells more efficiently, their gene silencing effects were weaker than those of PS AONs when tested using the same model assay. It seems that the conversion of the modified ONs into native ONs might occur too slowly inside cells to improve gene silencing.

#### Modifications at the sugar 2’-OH

**Scheme 2:**
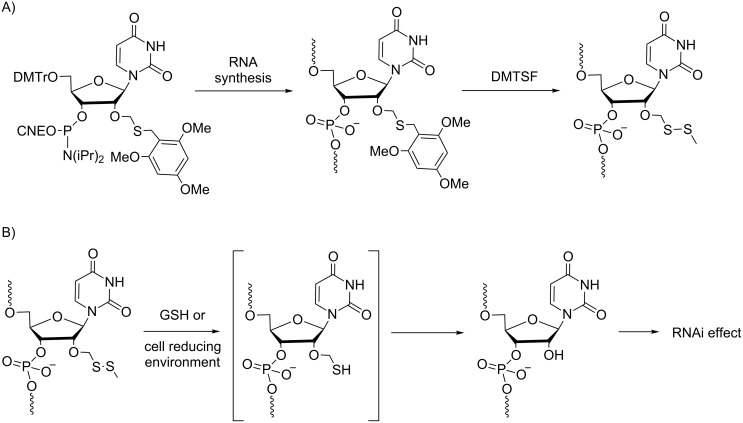
A) Synthesis via phosphoramidite chemistry and B) demasking under the reducing environment of 2’-*O*-MDTM-modified siRNA prodrugs [17].

Several permanent 2’-O-modifications (2’-F, 2’-OMe) have been proposed to increase the nuclease resistance of ONs, but most of them have decreased gene silencing potential. To overcome this drawback, novel prodrug-type RNAs containing a disulfide bridge at the 2’-position have been designed, and in 2016, Urata and our group reported on the synthesis and properties of 2’-*O*-alkyldithiomethyl-modified RNAs [15–16]. Previously, Urata had described a post-synthetic approach for the synthesis of 2’-*O*-methyldithiomethyl (MDTM) ONs [17] that was more practical than the phosphoramidite approach used initially for the chemical synthesis of RNAs using the 2’-*O-tert*-butyldithiomethyl-protecting group [18]. In the recent approach, the MDTM modification was obtained in excellent yield after conversion of the 2,4,6-trimethoxybenzylthiomethyl precursor group by treatment with dimethyl(methylthio)sulfonium tetrafluoroborate (DMTSF, Scheme 2). First, ONs containing 2’-*O*-MDTM modifications have shown greater nuclease resistance, and they were rapidly and efficiently converted into 2’-OH ONs under reducing conditions (10 mM 1,4-dithiothreitol or 10 mM glutathione, pH 7) [17]. In a subsequent report [16], the unmasking of double-stranded 2’-*O*-MDTM siRNAs into 2’-OH siRNAs was similarly demonstrated in the presence of 10 mM GSH. Furthermore, firefly luciferase expression in A549-Luc cells was inhibited by 2’-*O*-MDTM siRNAs to a higher extent than the unmodified siRNA regardless of the modification site (5’-end and/or the seed region of the antisense strand). These results suggest that 2’-*O*-MDTM siRNAs fulfill some features of typical prodrug-type siRNAs.

**Scheme 3:**
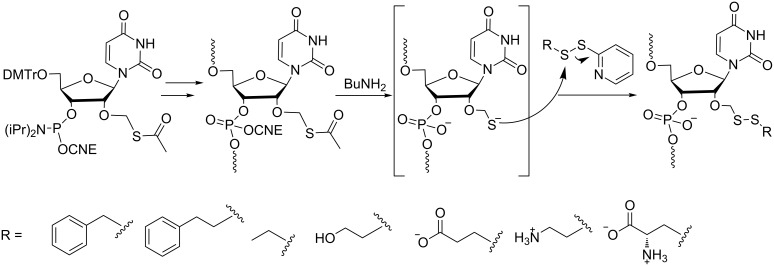
Synthesis via phosphoramidite chemistry of various 2’-*O*-alkyldithiomethyl (RSSM)-modified RNAs bearing lipophilic or polar groups (R) involving post-elongation conjugation through a thiol disulfide exchange reaction [15].

Similarly, our group has developed a post-synthetic method on a solid support to introduce various disulfide bond-containing groups at the 2’-OH of RNAs [15]. Using this versatile method, one precursor, 2’-*O*-acetylthiomethyl-containing RNA, produces various 2’-*O*-alkyldithiomethyl (RSSM)-modified RNAs bearing lipophilic or polar groups through a thiol disulfide exchange reaction with alkyldisulfanyl-pyridine derivatives (Scheme 3). In a preliminary evaluation, the RSSM modifications were shown to increase RNA resistance against 3’-exonuclease and not disturb the duplex stability too much while maintaining an A-form conformation. In addition, glutathione treatment under physiological conditions rapidly and efficiently reduced all the RSSM groups releasing 2’-OH RNA. These properties are promising for the use of 2’-*O*-RSSM-modified RNAs as prodrugs of siRNAs.

#### Modifications at the extremities

**Scheme 4:**
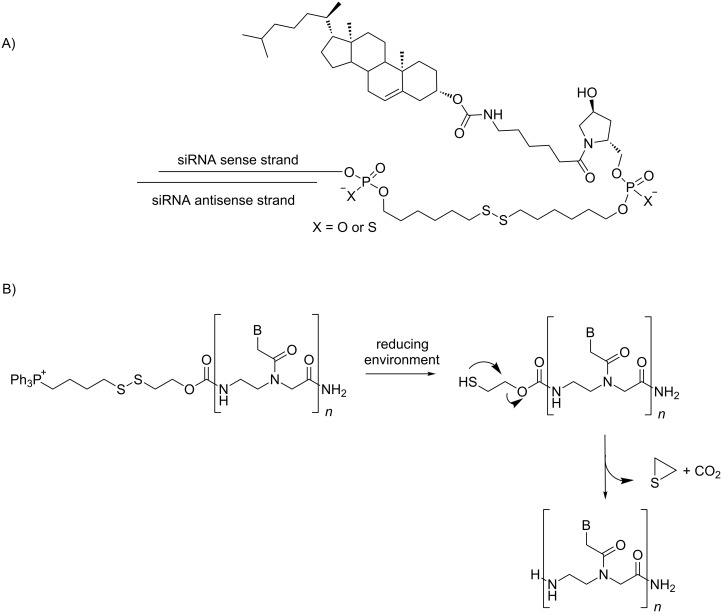
A) siRNA conjugates to cholesterol [19] and B) PNA conjugates to a triphenylphosphonium [20] through a disulfide linkage.

Disulfide bonds are attractive in designing drug-delivery systems. Indeed, lipophilic moieties may be attached to ONs to enhance cellular uptake. In particular, a cleavable disulfide linker has been used at the 3’-end of the sense strand to prepare cholesterol-conjugated siRNAs that were efficiently delivered to rat oligodendrocytes in vivo and achieved significant specific gene knockdown in these cells (Scheme 4A) [19]. The comparison with a non-cleavable alkyl linker suggests that a lipophilic siRNA conjugate with a disulfide linker is favorable to improve the suppression of 2’,3’-cyclic nucleotide 3’-phosphodiesterase mRNA in oligodendrocytes in vivo. This result may be attributable to increased bioavailability of siRNA in the cytoplasm.

Similarly, regarding the intracellular delivery of naked peptide nucleic acids (PNAs), a lipophilic triphenylphosphonium (TPP) cation was attached to the N-terminal extremity of a PNA through a biodegradable carbamate linker containing a disulfide bridge (Scheme 4B) [20]. It was shown that such PNA conjugates entered cells rapidly and efficiently. Furthermore, a 16-mer PNA_TAR_ fragment directed against the TAR RNA region of the HIV genome conjugated to TPP inhibited HIV replication in CEM cell lines with an IC_50_ of 1 μM, while the unconjugated 16-mer PNA_TAR_ was inactive in these tests. The anti-HIV activity confirmed that the PNA_TAR_ was not sequestered in mitochondria; consequently, the disulfide bond was reduced into the cytoplasm.

### Enzyme-responsive ONs

A control of gene expression using cellular enzymes as triggers of the activity of ON prodrugs is very attractive because this approach is based on the difference in the extra- and intracellular contents of the enzymes. Therefore, the biodegradable modification present in the prodrug could not be removed in extracellular media but only inside the cells. Two approaches have been reported using reductases or carboxyesterases to trigger transformation of ON prodrugs in native ONs. Although a post-synthesis introduction of the enzymolabile groups into phosphorothioate ONs by the reaction with alkyl iodides has been considered since the mid 90's [21–24], the use of phosphoramidite building blocks bearing the enzymocleavable group is the method of choice for synthesizing ON prodrugs regardless of the protected function (phosphate, nucleobase, sugar hydroxy groups).

#### Reductase-responsive ONs

Hypoxic conditions that are characteristic of solid tumors represent a remarkable stimulus to convert non-active prodrugs into active drugs under reductase action. Three examples of hypoxia-activated ONs have been reported thus far, with a hypoxia-labile modification either in the phosphate backbone to mask the negative charge and provide better tumor selectivity [25–26] or at the nucleobase to modulate the hybridization properties with the target [27]. In all cases, a nitro-derivative-modified thymidine phosphoramidite was prepared and incorporated into oligothymidylates (dT)*_n_* or heterosequences at different sites. Actually, the nitro-derivative modifications (nitrobenzyl, nitrofuryl or nitrothienyl) can be reduced by reductases to form the corresponding amino (or hydroxylamino) derivatives, followed by a cleavage of the benzyl or heterocycle groups and release of the unmodified sequences.

**Scheme 5:**
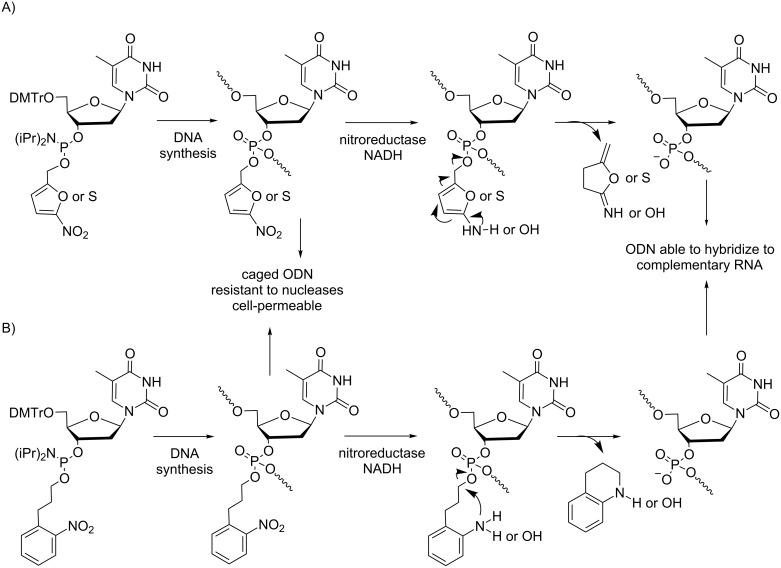
Synthesis via phosphoramidite chemistry and deprotection mediated by nitroreductase/NADH of hypoxia-activated prodrugs of ONs containing A) 5-nitro-2-furylmethyl or 5-nitro-2-thiophenylmethyl [25] and B) 3-(2-nitrophenylpropyl)phosphotriester internucleoside linkages [26].

**Modifications at the internucleotide linkage:** ONs containing either 5-nitro-2-furylmethyl or 5-nitro-2-thiophenylmethyl modifications at some internucleoside phosphates were converted to native (dT)*_n_* with good hypoxia selectivity in vitro by nitroreductases as well as in tumor cell extract by cellular reductases (Scheme 5A) [25]. Furthermore, such nitrofuryl and nitrothienyl modifications improved nuclease resistance and cellular uptake of ONs in proportion to the number of lipophilic groups. In another study, a series of ONs with mixed sequences bearing some nitrophenylpropyl modifications were synthesized and exhibited good resistance toward nucleases and stability in human serum (Scheme 5B) [26]. Their cellular uptake in HeLa cells was greater than that of the naked ON and increased with the number of labile groups masking the phosphates. As expected, the nitrophenylpropyl groups were readily cleaved by nitroreductase in the presence of NADH. Such modified ONs could be used as prodrugs for the delivery of ON-based therapeutics in hypoxic cells.

**Scheme 6:**
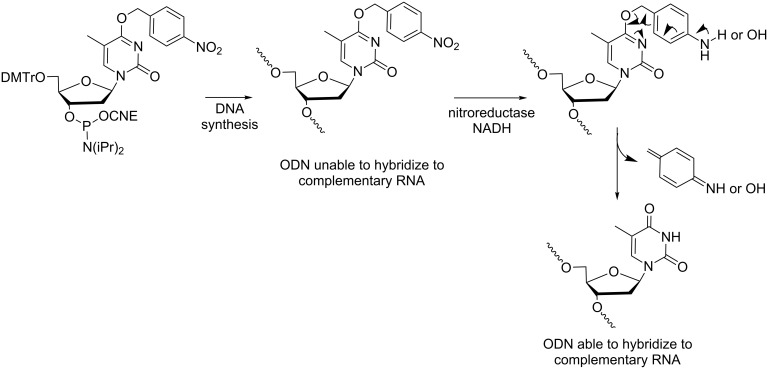
Synthesis via phosphoramidite chemistry and conversion mediated by nitroreductase/NADH of hypoxia-activated prodrugs of ONs containing *O*^4^-(4-nitrobenzyl)thymidine [27].

**Modifications at the nucleobase:** The third example reported by Saneyoshi and Ono refers to ONs containing the hypoxia-labile group on the nucleobase. It was shown that (dT)_5_ with one 4-nitrobenzylthymine was deprotected in vitro by nitroreductase in the presence of NADH to produce (dT)_5_ with native thymine (Scheme 6) [27]. In addition, thermal stabilities of the duplexes formed with thymine-modified ONs and their complementary sequences were evaluated; the nucleobase modifications induced an important destabilization of the duplexes. This result suggests that 4-NO_2_-benzylthymine-modified ONs cannot hybridize to their targets and consequently should be inactive in normal cells. However, in hypoxic cells after removal of the 4-nitrobenzyl groups, the resulting native ONs should form stable active duplexes with their targets. These hypoxia-labile modifications seem promising for the development of ON therapeutics with specific activity in hypoxic tumor cells and low toxicity in normal cells.

**Scheme 7:**
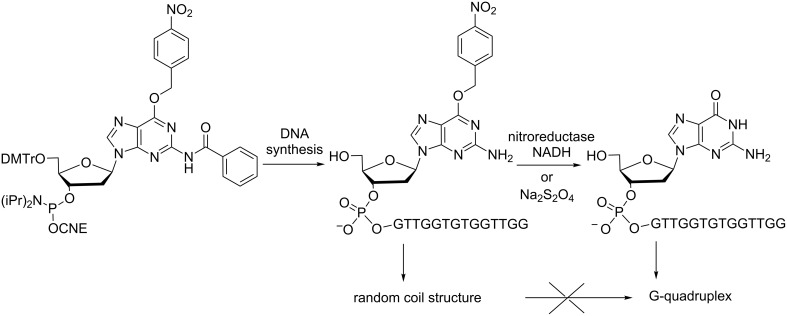
Incorporation of *O*^6^-(4-nitrobenzyl)-2’-deoxyguanosine into an ON prone to form a G-quadruplex structure, preventing it from forming this quadruplex when protected and allowing it under reducing conditions [28].

A nitrobenzyl (NB) group has also been introduced at O6 of a guanine to modulate the conformational properties of a G-quadruplex structure-forming single-stranded DNA [28]. The dG^NB^ phosphoramidite was synthesized and incorporated into the sequence of a thrombin-binding DNA aptamer (TBA, at the 5’-end) prone to form a G-quadruplex structure (Scheme 7). Circular dichroism studies have indicated that TBA^NB^ adopts a random coil structure while after reduction caused by chemical (Na_2_S_2_O_4_) or enzymatic (nitroreductase with NADH) stimuli, the formation of a G-quadruplex structure was evidenced due to the conversion of TBA^NB^ into TBA. The modulation of the secondary structure transition of an ON in a reduction-responsive manner appears to be beneficial to understand biomolecule behavior and biological phenomena.

#### Esterase-responsive ONs

**Scheme 8:**
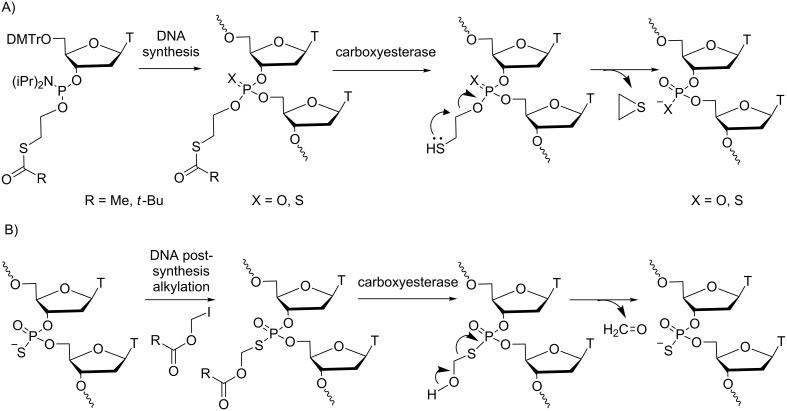
Synthesis and mechanism for the demasking of ON prodrugs from A) *S*-acylthioethyl phosphotriester [29] and B) *S*-acyloxymethyl phosphotriester [22].

**Modifications at the internucleotide linkage:** The use of phosphate modifications cleaved under carboxyesterase mediation was envisaged for ONs more than 20 years ago and was extensively studied by Imbach’s group [29] and others [22,30–31]. Ten years ago, Lönnberg summarized the chemical aspects of prodrug strategies at the nucleotide and oligonucleotide levels and particularly focused on esterase-responsive modified-phosphate ONs [32]. The most studied masking groups have been the methyl-SATE (*S*-acetylthioethyl) and *tert*-butyl SATE (*S*-pivaloylthioethyl) developed by Imbach (Scheme 8A) [29], whereas *S*-acyloxymethyl groups were studied by Agrawal (Scheme 8B) [22]. The fundamental advantage of using enzyme-cleavable modifications of the phosphodiester backbone in ONs is to transitorily mask the negative charges of the phosphate by neutral phosphotriesters. Consequently, the backbone is less prone to nuclease degradation, and the lipophilicity of the pro-ON increases cell permeation [33]. The uptake was proportional to the number of SATE groups and probably proceeded through a passive diffusion mechanism [34]. Furthermore, it was shown that SATE-protected phosphates were selectively demasked in cell extracts [35–37]. SATE thionophosphotriester ONs were quantitatively converted to phosphorothioate ONs by carboxyesterase-mediated deacylation followed by the removal of the resulting *S*-(2-mercaptoethyl) group by cyclization to episulfide. For *S*-acyloxymethyl phosphorothiolates, hydrolysis of the ester catalyzed by the enzymes was followed by release of formaldehyde to produce the phosphorothioate ON.

**Figure 1:**
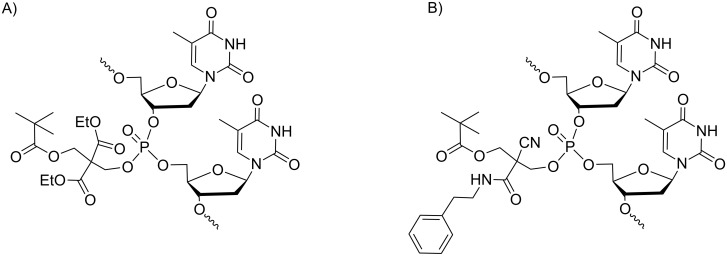
Oligothymidylates bearing A) 2,2-bis(ethoxycarbonyl)-3-(pivaloyloxy)propyl- and B) 2-cyano-2(2-phenylethylaminocarbonyl)-3-(pivaloyloxy)propyl phosphate protecting groups [41].

Despite these promising results, further studies on the use of these prodrugs to control genetic expression have not been carried out. Thus far, most of these results were obtained for thymidine homopolymers [32]. The reason is that the synthesis of ON prodrugs is incompatible with the standard deprotection treatment under basic conditions (generally aqueous ammonia) used to cleave other common base-labile acyl protection groups from nucleobases and release ON from the solid support. Furthermore, as the aqueous solubility of fully modified SATE phosphotriester ONs is rather poor [29], the design of ONs combining phosphodiester and phosphotriester linkages is required to ensure aqueous solubility and sufficient lipophilicity for cell uptake. Several attempts to obtain such chimeras were made in Imbach’s laboratory in the early 2000s. In particular, the use of photolabile protecting groups [38] of allyloxycarbonyl groups deprotected by Pd(0) [39] and of fluoride-labile groups [40] in place of the standard acyl protection of nucleobases has made possible the acquisition of short sequences of heteropolymer pro-oligonucleotides. However, none of these methods led to ON prodrugs of therapeutic interest in the antisense approach. A similar conclusion can be drawn from Lönnberg's work reported in 2005 that described the synthesis of homothymidylates and phosphorothioate analogs protected by the biodegradable 2,2-bis(ethoxycarbonyl)-3-(pivaloyloxy)propyl and 2-cyano-2-(2-phenylethylaminocarbonyl)-3-(pivaloyloxy)propyl groups (Figure 1A and 1B) [41]. Indeed, this work also did not lead to ONs for use in control of gene expression.

**Figure 2:**
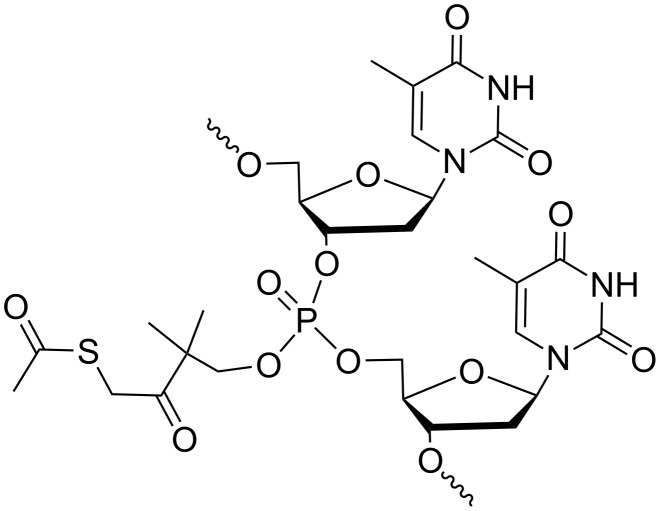
Oligothymidylates containing esterase and thermo-labile (4-acetylthio-2,2-dimethyl-3-oxobutyl) phosphate protecting groups [42].

In addition, Lönnberg described the 4-acetylthio-2,2-dimethyl-3-oxobutyl group as another phosphate protecting group that should be removed by both, esterases and heat (Figure 2) [42]. The resulting phosphotriesters of short oligothymidylates were successfully converted into phosphodiesters at 37 °C, but some cleavage of internucleosidic bonds also occurred. The slow conversion could be accelerated upon the addition of hog liver esterase, but the accumulation of negative charge slowed down the enzymatic hydrolysis. These preliminary data did not provoke further development of such an approach.

**Scheme 9:**
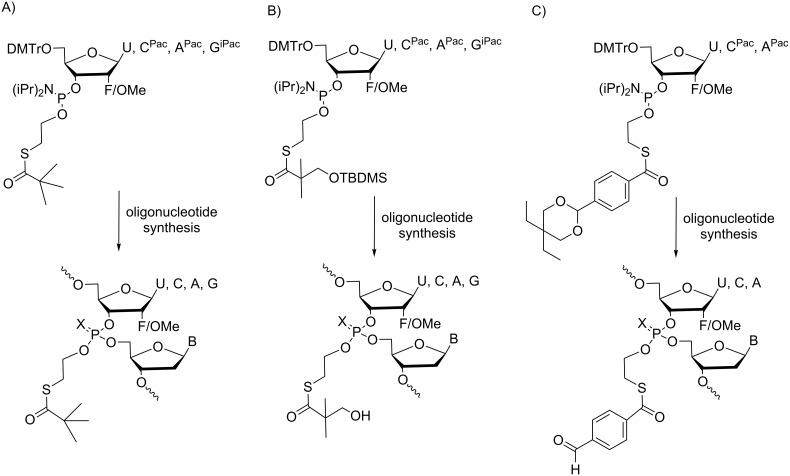
Phosphoramidites and the corresponding RNA prodrugs protected as A) *t-*Bu-SATE, B) OH-SATE and C) A-SATE phosphotriesters [43].

Unfortunately, despite many strategies, all attempts to synthesize DNA ONs with SATE-phosphotriesters resulted in poor synthetic yields that made biological evaluation impossible. Consequently, for about ten years, research in the field of carboxyesterase-responsive ONs protected at the phosphate backbone had waned until Dowdy reported on the synthesis, delivery and in vivo activity of siRNA prodrugs containing charge-neutralizing phosphotriester linkages [43]. This recent publication, which was twice highlighted by C. Ducho [44] and A. Khvorova [45], is a reference in the field of ON prodrugs because, for the first time, a biological effect was measured in mice. Indeed, Dowdy’s group succeeded in the synthesis of a library of more than 40 phosphotriester groups on ribonucleic neutral (RNN) phosphoramidite building blocks containing 2’-modifications (2’-F, 2’-OMe) to avoid 2’-OH nucleophilic attack on the phosphotriester linkage. Moreover, they used extremely mild basic diisopropylamine in methanol to deprotect nucleobases containing phenoxyacetyl (for A and C) or isopropylphenoxyacetyl (for G) groups on exocylic amines. These deprotection conditions prevent base-mediated phosphotriester cleavage. Finally, to address the synthetic issue completely, they stabilized the thioester bond to diisopropylamine/methanol by substituting electron-donating groups at the distal α-carbon or lengthening the proximal ethyl linker to a butyl linker. With such RNN phosphoramidite building blocks >3000, RNN ONs have been synthesized with high yields comparable to those of RNA synthesis, demonstrating the robustness and versatility of the chemical method. Three enzymolabile phosphotriester groups, namely, *t-*Bu-SATE, OH-SATE and a conjugable aldehyde A-SATE for conjugation to delivery and targeting domains, have been selected for complete evaluation (Scheme 9A, 9B, and 9C, respectively). The optimum phosphotriester placement and number of phosphotriester groups were shown to have an important impact on the siRNA solubility and duplex stability. Such designed siRNNs showed a high solubility and serum stability and are not recognized by the innate immune system. On the other hand, due to their large size, they do not passively cross cell membranes. Therefore, to facilitate their uptake, a TAT-peptide delivery domain was conjugated to the siRNNs via A-SATE phosphotriester groups. Hence, a chimeric passenger strand containing four A-SATE phosphotriesters duplexed with an RNN guide strand was conjugated to the delivery domain TAT peptides. The resulting conjugates possessing only ≈25% of neutralized phosphates and four TAT peptides were optimal to enter cells passively. Once inside the cells, the SATE groups were efficiently removed by esterases, leading to siRNAs that are induced according to knockdown with apparent EC_50_ values in the low nanomolar range and in a noncytotoxic fashion. Next, the authors prepared conjugates of the siRNNs via one A-SATE phosphotriester with a hepatocyte-specific tris-*N*-acetylgalactosamine targeting domain and demonstrated a stronger RNAi response in mouse liver (following subcutaneous or intravenous administration) than the same conjugates with non-enzymolabile phosphotriesters as reference compounds. In conclusion, from this relevant study, it is noteworthy that for the first time, siRNA prodrugs have been synthesized by a versatile method and are intracellularly converted into natural phosphodiester siRNAs that induce robust RNAi responses in vivo. This work clearly opens the way to the new development of ON prodrugs for RNAi therapeutics.

**Scheme 10:**
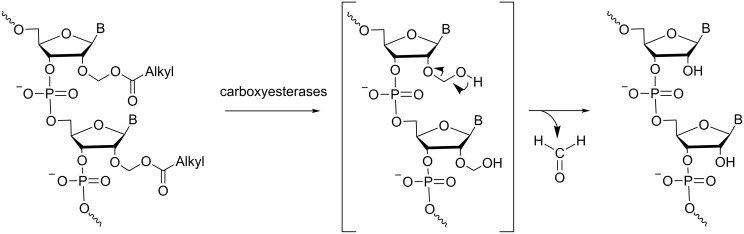
Mechanism of the hydrolysis of 2’-*O*-acyloxymethyl ONs mediated by carboxyesterases [46]. The hydrolysis of the ester functions yields an unstable 2’-hemiacetal, affording the free RNA through the release of formaldehyde.

**Modifications at the sugar:** For the last ten years, our group has been more interested in making RNA prodrugs with enzyme-cleavable modifications at the 2’-position. We essentially focused on several acetalester groups whose lipophilicities and stabilities were variable to tune siRNA properties, particularly their delivery. The first evaluation of biolabile 2’-O-modifications was achieved using short oligo-U sequences containing 2’-*O*-acyloxymethyl or acylthiomethyl groups [46–47]. They were shown to improve RNA nuclease resistance and not to hamper duplex dsRNA formation, and they are removed by cellular esterases. Indeed, 2’-*O*-acyloxymethyl ONs are converted to unmodified RNAs by carboxyesterase-mediated deacylation with the release of formaldehyde to produce the parent RNA (Scheme 10).

**Scheme 11:**
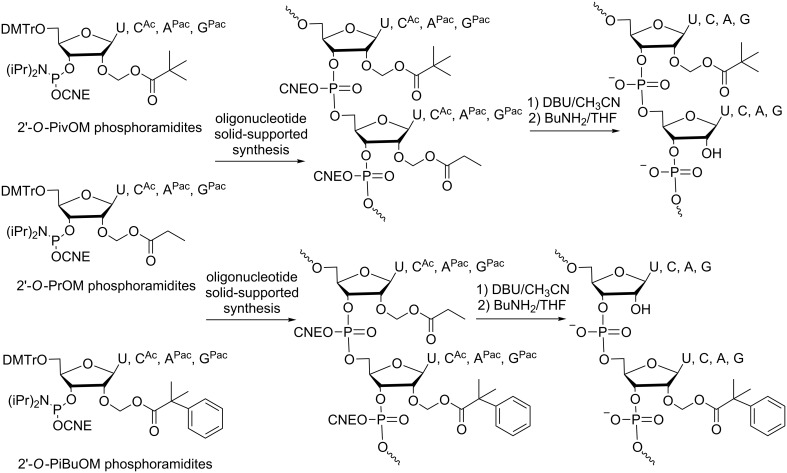
Synthesis of partially 2’-*O*-PivOM-modified RNAs [49] and 2’-*O*-PiBuOM-modified RNAs [53] using their corresponding phosphoramidites and 2’-*O*-PrOM phosphoramidites to generate 2’-OH.

These features made 2’-*O*-acetalester modifications promising for their use in a prodrug approach; of particular interest was the pivaloyloxymethyl (PivOM) group, which completes the requirements to functionalize a potential siRNA prodrug. Therefore, for the first time, several mixed-nucleobase RNAs partially 2’-O-masked with PivOM groups were synthesized via a solid-phase method involving silyl-based protections on amino functions of the nucleobases combined to CNE on phosphates and Q-linker between pro-RNA and the solid support [48]. One of them with five PivOM groups at the 5’-end was active in a human cell culture-based RNA interference assay, and it exerted improved cellular uptake. These preliminary data provided a proof-of-concept for a prodrug-based approach for the delivery of siRNA to living human cells. The next report described a more convenient and straightforward method to synthesize partially modified 2’-*O*-PivOM RNAs (Scheme 11) [49]. The strategy involves standard labile acyl groups for nucleobases, cyanoethyl groups for phosphates, a Q-linker to the solid support [50] and two acetal ester groups for 2’-OH, namely, propionyloxymethyl (PrOM) and PivOM exhibiting different stability under deprotection conditions. Indeed, a specific treatment with butylamine in anhydrous THF [51] selectively removes the PrOM groups while the PivOM groups stay attached. Thus, partially PivOM-modified siRNAs with a different design have been evaluated. No serious thermal destabilization of the siRNA duplex was observed and the A-form duplex was maintained [52]. Moreover, all PivOM-modified siRNAs (1 nM) showed control of gene expression activity after transfection into ECV304 cells expressing the firefly luciferase gene. Nevertheless, the RNAi activity of such 2’-*O*-acetal ester siRNAs taken up by cells in the absence of any carriers remained to be demonstrated. The robust synthetic method developed in 2014 [49] made 2’-PivOM-modified siRNAs readily available. To improve their lipophilic features, one methyl of the *tert*-butyl moiety in the PivOM groups was replaced by one phenyl, resulting in the phenylisobutyryloxymethyl (PiBuOM) modification, which was introduced into siRNAs for investigation (Scheme 11) [53]. Indeed, we provided evidence of improved spontaneous cellular uptake of naked PiBuOM-modified siRNAs compared to unmodified or PivOM-modified siRNAs. Consequently, a substantial inhibition (90% at 1 μM concentration) of EWS-Fli1 expression in A673 cells in serum-containing medium was observed. It is noteworthy that this PiBuOM modification is efficient in assisting siRNAs to enter cells and promote gene inhibition without the use of transfecting agents. Furthermore, even if the intended prodrug strategy was not validated with PiBuOM modification because of a certainly too slow esterase cleavage, its use in the sense strand as permanent lipophilic modification has been relevant to facilitating the cellular uptake of siRNAs and subsequent gene inhibition.

**Figure 3:**
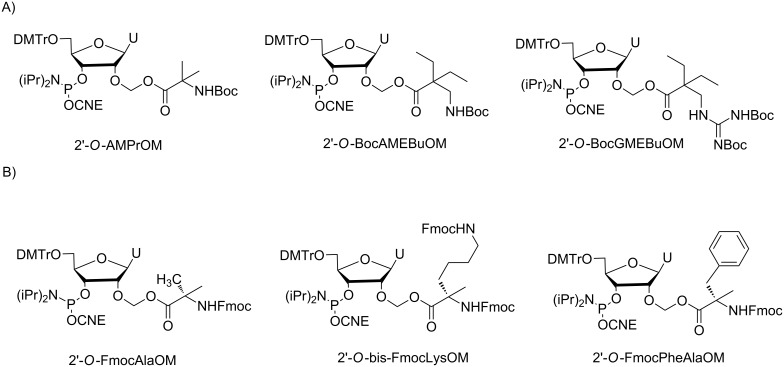
A) 2’-*O*-amino and guanidino-containing acetal ester phosphoramidites and B) 2’-*O*-(amino acid) acetal ester phosphoramidites reported by Debart [54] and Dahma [55], respectively.

Beside it is known that cellular internalization properties can be improved by adding positive charges to ONs to counterbalance the overall negative charge of these compounds. In this context and in extension of the previous work with the 2’-*O*-acetal ester modifications cited above, new modified ONs were designed with amino or guanidino-containing 2’-*O*-acetal ester groups bearing positive charges: 2-amino-2-methylpropionyloxymethyl (AMPrOM), 2-aminomethyl-2-ethylbutyryloxymethyl (AMEBuOM) or 2-guanidinomethyl-2-ethylbutyryloxymethyl (GMEBuOM, Figure 3A) [54]. The two modifications with a guanidinium and an ammonium moiety, GMEBuOM and AMPrOM, respectively, were found to be unstable during HPLC purification and handling. Therefore, they could not be further investigated. By contrast, the AMEBuOM modification was evaluated within several 2’-OMe ONs or a fully AMEBuOM-modified ON, which was more resistant to enzymatic degradation. A slightly moderate internalization of AMEBuOM-modified ON (ammonium side chain) was observed compared to the ON with the PivOM group (*t-*Bu side chain), probably due to the instability of AMEBuOM groups in cell culture medium before internalization. Overall, these cationic acetal ester modifications are chemically too unstable for further developments as ON prodrugs. Similarly, Damha reported on the synthesis of ONs containing amino acid-acetal esters at the 2’-OH, particularly with lysine for its positive charge (Figure 3B) [55]. Unfortunately, 2’-*O*-acetal ester ONs with lysine, alanine and phenylalanine could not be isolated with good yield because they were partially degraded during HPLC purification and subsequent handling. No further study has been described in the literature with such 2’-modified ONs.

**Scheme 12:**
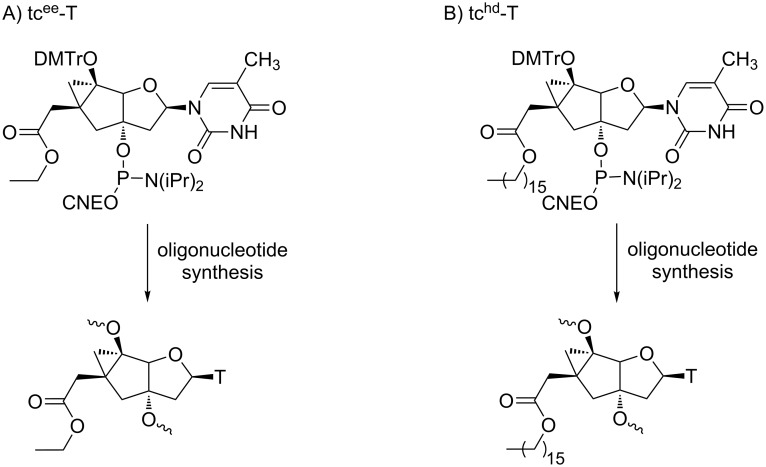
Prodrugs of tricyclo-ONs functionalized with A) ethyl (tc^ee^-T) and B) hexadecyl (tc^hd^-T) ester functions at C6 obtained from corresponding thymidine phosphoramidites [56].

Prodrugs of conformationally constrained nucleic acids such as tricyclo-DNA (tc-DNA) deserve to be mentioned in this review as sugar-modified ONs. Indeed, tc-DNAs were evaluated as promising candidates for ON-based therapeutic applications, exhibiting increased affinity to RNA and better resistance to nucleases. The main bottleneck of their use, as for many other modified ONs, is their poor cellular uptake. Therefore, to address this issue, Leumann et al. synthesized “pro-tricyclo-ONs” bearing two different metabolically labile ethyl and hexadecyl esters at position C6’ that were expected to promote cell penetration (Scheme 12) [56]. It was shown that the cellular uptake of a decamer containing five tc^hd^-T units with a C_16_ side chain was increased in two different cell lines (HeLa and HEK 293T) without using a transfection agent. Nevertheless, the enzymatic hydrolysis of the hexadecyl esters and some preliminary antisense activities remain to be demonstrated.

### Heat-responsive ONs

These so-called ONs contain thermolytic groups that are removed upon a ‘heat-driven’ process under neutral conditions.

#### Modifications at the internucleotide linkage

**Scheme 13:**
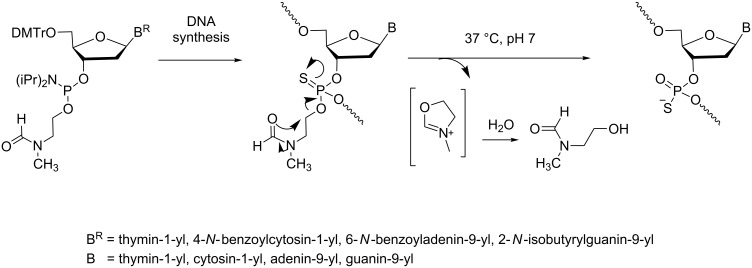
Demasking mechanism of *fma* thiophosphate triesters in CpG ODN upon heat action [58].

Over many years, various thermolytic groups for 5’-OH and phosphate protections have been designed and developed by Beaucage et al. to synthesize DNA ONs on microarrays due to their rapid removal under mild conditions [57]. Heat-sensitive phosphate/thiophosphate-protecting groups have been incorporated into ONs via phosphoramidite chemistry using solid-support methodology. However, some required more drastic conditions (90 °C for a long period of time) to be cleaved, and Beaucage found a potential application of such thermolytic ONs as prodrugs in the treatment of infectious diseases. Even if in this review, the applications of ON prodrugs are essentially focused on gene silencing, it seemed important to us to report on the thermolytic CpG-containing ODNs as potential immunotherapeutic prodrugs [58]. The first impressive result was obtained in vivo with a CpG ODN (CpG ODN *fma*1555) functionalized with the 2-(*N*-formyl-*N*-methyl)aminoethyl (*fma*) thiophosphate protecting groups, which were cleaved at 37 °C to yield the well-known immunomodulatory CpG ODN 1555 (Scheme 13). When the CpG ODN *fma*1555 was administrated to newborn mice that had been infected with Tacaribe virus, 43% of mice survived [58]. Moreover, an improved immunoprotection (60–70% survival) was obtained when the CpG ODN prodrug was administered three days before infection. Interestingly, it also was shown that the combination of CpG ODN 1555 and CpG ODN *fma*1555 (more than 50% survival) increased the window for therapeutic treatment against the disease. However, the induction of the immunostimulatory effect was delayed, which is consistent with the formation of the biologically active phosphorothioate diesters from the *fma* thiophosphate triesters with a thermolytic conversion half-life of *t*_1/2_ = 73 h at 37 °C.

**Scheme 14:**
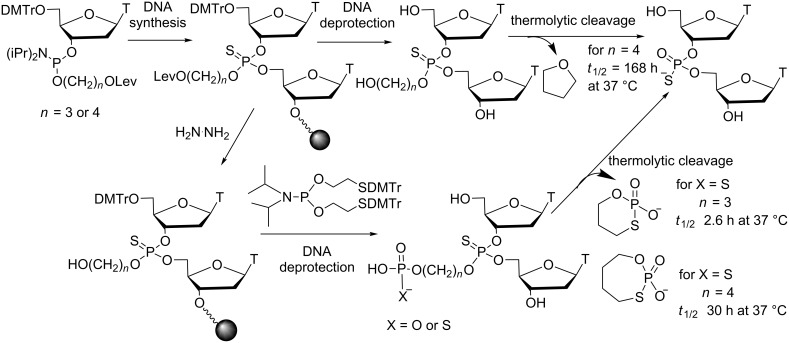
Thermolytic cleavage of the hydroxy-alkylated thiophosphate and phosphato-/thiophosphato-alkylated thiophosphate protecting groups from thymidine dinucleotides [59].

Although these *fma* ODNs exhibit the features of ON prodrugs in that they are neutral to enable cellular delivery and are stable to hydrolytic nucleases, Beaucage et al. developed other thermolytic ONs with thermolabile groups displaying slower or faster removal kinetics than that of *fma* groups. In particular, the subsequent heat-sensitive groups for phosphate masking were designed with a phosphate or a thiophosphate branched to a propyl or a butyl chain connected to the internucleoside linkage (Scheme 14) [59]. Consequently, the presence of only one phosphate monoester function in an *fma* ON significantly increased the solubility. Unfortunately, no biological evaluation of such modified ONs was performed, and only the complete conversion of modified CpG into unmodified CpG upon elevated temperature conditions was shown.

Another study in the same laboratory described new heat-sensitive thiophosphate protecting groups derived from the previously cited *fma* [58] and 4-(methylthio)butyl groups [57]. Some 20 groups, which will not be detailed here, have been assessed and were found to exhibit slower or faster thermolytic deprotection rates than those of the *fma* group at 37 °C (*t*_1/2_ = 72 h) [60]. Typically, the thermostable groups with deprotection kinetics slower than those of the *fma* group may be used for the protection of terminal phosphodiesters of the immunomodulatory DNA sequence targeting the nuclease resistance of the ON prodrug. On the other hand, the thermosensitive groups are more suitable for the protection of the thiophosphates flanking the CpG motif of DNA prodrugs to provide both lipophilicity (better cellular uptake) and hydrophilicity (better solubility once groups are removed). Moreover, some of thermolabile groups (*t*_1/2_ in the range of 6 h to 40 h at 37 °C) may be applicable to protect the thiophosphates of CpG motifs of immunoregulatory DNA sequences. Thus, the investigation of these different heat-sensitive groups may serve to design optimal CpG DNA prodrugs.

**Scheme 15:**
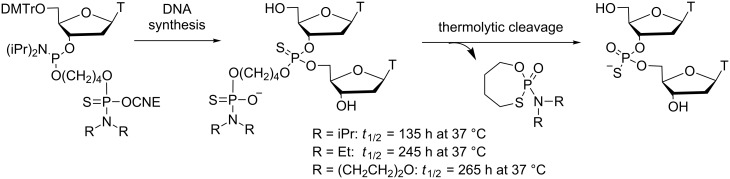
Synthesis via phosphoramidite chemistry and thermolytic cleavage of alkylated (diisopropyl, diethyl, morpholino) phosphoramidothioylbutyl internucleoside linkages [61].

Similarly, in the search for thiophosphate protecting groups with deprotection half-lives in the range of 100–200 h at 37 °C for sustained CpG ODN immunostimulation in animal models, Beaucage et al. have developed a new class of thermosensitive groups that are hydroxy-alkylated phosphoramidate, phosphoramidothioate and phosphorodiamidothioate derivatives (Scheme 15) [61]. Their thermolytic deprotection rates at 37 °C have been determined in PBS (pH 7.4) from thymidine dinucleoside phosphorothioate models. It was shown that the thermolytic cleavage of alkylated (diisopropyl, diethyl, morpholino) phosphoramidothioylbutyl groups to TpsT proceeded with respective half-lives of 135 h, 245 h and 265 h at 37 °C. Therefore, these groups are appropriate for thiophosphate protection of the CpG motif of CpG ODN prodrugs, and they are complementary to those identified earlier [60]. It remains to study such thermosensitive CpG ODNs in animal models infected by viruses and/or bacteria to evaluate the correlation between extended immunostimulation and resistance.

**Scheme 16:**
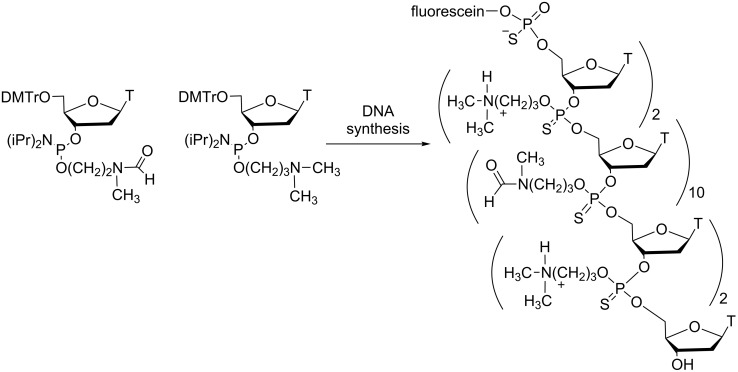
Synthesis of thermosensitive prodrugs of ODNs containing *fma* thiophosphate triesters combined to positively charged 3-(*N,N*-dimethylamino)propyl phosphotriesters internucleoside linkages to improve cellular uptake [62].

The most recent data reported by Beaucage on thermosensitive PS DNA prodrugs were related to the assessment of their internalization in various cell lines [62]. The study was essentially performed with oligothymidylate models. First, the internalization of a 5’-fluorescein *fma* (Tps)_14_T in Vero, HeLa and GC-2 cells was poor but comparable to that of the control 5’-fluorescein (Tps)_14_T. These data can be explained by the decreased solubility in aqueous medium of the uncharged ON and can be correlated with the similar abilities of CpG ODN *fma*1555 and CpG ODN 1555 to induce an immunostimulatory response in the mice mentioned above [58]. On the other hand, the introduction of four positively charged 3-(*N,N*-dimethylamino)propyl groups into an *fma*-thiophosphate oligothymidylate resulted in enhanced aqueous solubility and a 40-fold increase in the cellular uptake of the ON in Vero and GC-2 cells (Scheme 16). It is noteworthy that the presence of four positively charged groups into a negatively charged PS oligothymidylate is not sufficient for an efficient cellular internalization in Vero cells. These data support that both 3-(*N,N*-dimethylamino)propyl and *fma* groups are required for optimal internalization in the three cell lines. Of special interest was the absence of cytotoxic effects in Vero cells at a 50 μM extracellular ON concentration for 72 h. Moreover, confocal microscopy studies showed that the positively charged oligoT escaped endosomal vesicles and migrated to the nucleus of Vero or GC-2 cells. This observation may support the correlation between cellular uptake and the activity of thermosensitive DNA prodrugs. Supplementary experiments with mixed-nucleobase DNA sequences should provide more information on these thermosensitive ON prodrugs.

Finally, it should be mentioned that additional thermolabile protecting groups for phosphodiesters have been reported by Lönnberg [63–64]. Actually, in the search for esterase-labile protecting groups for phosphoesters, a set of 2,2-disubstituted 4-acylthio-3-oxobutyl groups was additionally thermolabile. This investigation was only achieved at the nucleotide stage and no data with ONs were reported. Consequently, these special protecting groups will not be detailed in this review.

#### Modifications at the nucleobase

**Scheme 17:**
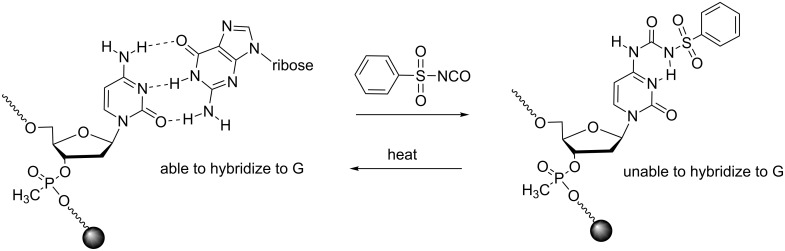
Caging of deoxycytidine in methylphosphonate ONs by using the thermolabile phenylsulfonylcarbamoyl protecting group introduced through reaction with phenylsulfonyl isocyanate [65].

The temporary protection of nucleobases by heat-responsive groups has not yet found applications in the field of ON prodrugs despite a certain potential. Indeed, the introduction of the phenylsulfonylcarbamoyl (psc) protection of cytosines in methylphosphonate ONs through the reaction with phenylsulfonyl isocyanate produces a caged ON unable to hybridize to its complementary RNA sequence until heat removal of the psc (Scheme 17) [65]. However, currently, this approach is limited to CPG-supported methylphosphonate ONs containing thymines and cytosines immobilized on a glass slide.

### Light-responsive ONs

Compared to other stimuli used to generate ONs that act as gene regulator, light is the external physical regulatory element that is most used. Actually, photoirradiation is the major and simplest method to temporally and spatially regulate the activity of photoresponsive ONs that could be assimilated to prodrugs, although this term is not commonly used except in a few reports [66]. Depending on the strategies used, the introduction of photolabile moieties into an ON renders it active or inactive and, therefore, it is turned on (light activated) or off (light deactivated) by light, respectively [67–71]. Thus, the advantages of light are to give the possibility of controlling this switch in time but also in space because photoirradiation could be performed only on a desired part of a sample, a cell, a tissue or a living organism. However, it should be noted that currently, most of the activities of photocaged ONs have been validated on reporter gene models except for a few studies on specific genes in zebrafish embryos.

Despite the advantages described above, the use of light to control gene expression has several drawbacks. Extended UV irradiation may produce side reactions, lowering the yield of active ON and inducing toxicity. Moreover, the diffusion of light resulting from long UV irradiation decreases temporal and spatial resolution for experiments in cells. Finally, because light has poor tissue diffusion, the photocaging approach may be restricted to in vitro gene-silencing interactions and of limited use for therapeutic applications.

#### Modifications at the phosphate moieties

The control of gene expression with photocaged phosphate-modified ONs has been mostly used for light activation of RNA interference, as commonly used by the Friedman group [72–76], and occasionally for RNA-cleaving activity with DNAzymes [77].

It is expected that phosphate-modified siRNAs sterically block the interaction of siRNA with the RISC complex and that the process is turned on upon photoirradiation [72]. Considering DNAzymes, their catalytic activity is inhibited until photoirradiation releases the native DNAzyme [77]. In phosphate-caged siRNAs, chemical derivatization of phosphates either in the phosphodiester backbone [72] or at a terminal phosphate [73–74] of ON was performed following two different approaches: a) post-functionalization of ON with a suitable reagent, which generally is a diazo derivative bearing a photoresponsive moiety, or b) incorporation of an appropriate photocaged phosphoramidite during the solid-supported ON synthesis [73,78]. The advantage of the first approach is that the functionalization results from a reaction with available unmodified ONs, while the second approach first requires the synthesis of a modified unit followed by its incorporation into ON during solid-phase synthesis. However, the first approach is far less efficient than the second one because the labeling of phosphodiester linkages with diazo compounds is not specific to a given phosphodiester in siRNA and cannot be controlled in location and the amount of caging units, yielding a random mixture of ONs. Moreover, diazo compounds exhibit certain reactivity toward nucleobases that can lead to undesired side reactions [74]. Considering their RNAi activity, these statistically phosphate-caged RNAs also have several drawbacks. Indeed, Friedman et al. have shown that low percentages of photolabile-protecting groups in siRNA only induce partial inhibition of gene silencing. Inversely, higher percentages increase the blocking of RNAi before light activation induces the release of photoresponsive moieties during photoirradiation, yielding a lower extent of GFP expression in HeLa cells [72].

**Figure 4:**
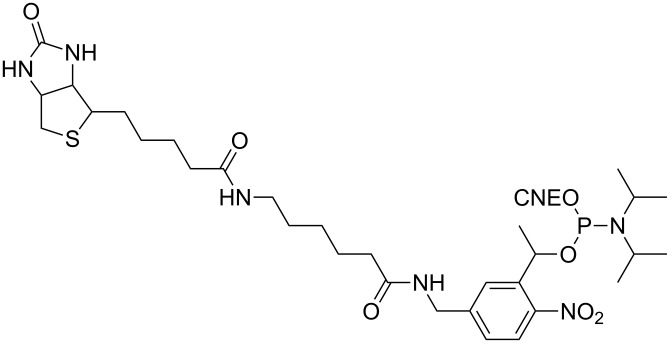
Biotinylated 1-(5-(aminomethyl)-2-nitrophenyl)ethyl phosphoramidite used to cage the 5’-end of a siRNA during its synthesis on solid support using phosphoramidite chemistry [73].

Later, Mc Master showed that it is not necessary to heavily modify siRNA because a single photoresponsive unit (biotin linked to nitrophenylethyl, Figure 4) at the phosphate located at the 5’-end of the antisense strand of a siRNA decreased RNAi, although only moderate photomodulated silencing of several transfected genes in HeLa cells was observed [73]. In this work, the responsive unit was introduced into an ON using the corresponding phosphoramidite (Figure 4), but Friedman showed that this also could be done by the reaction of diazo compounds with the terminal phosphates of an ON. Indeed, the reactivity of diazo reagents with terminal phosphates (phosphomonoesters) was much greater and more specific than that with the internucleoside phosphates (phosphodiesters) [74].

**Scheme 18:**
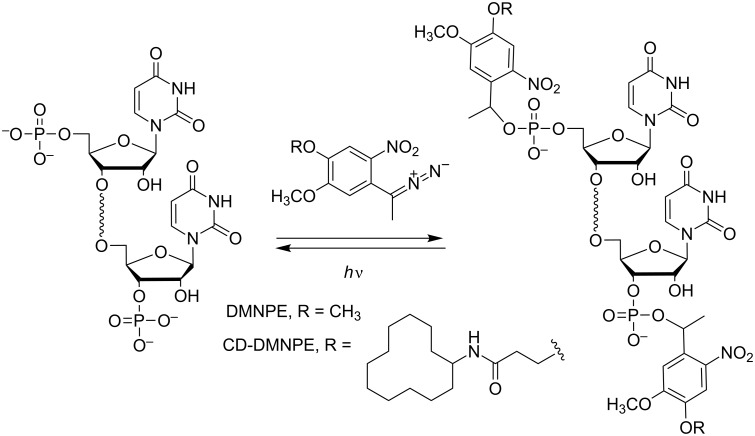
Introduction and cleavage of 1-(4,5-dimethoxy-2-nitrophenyl)ethyl (DMNPE) [74] and cyclododecyl-DMNPE (CD-DMNPE) [76] groups in the terminal 3’ and 5’-phosphate of an RNA through reaction with a diazo reagent.

Friedman improved the efficiency of the phosphate caging approach by introducing photolabile moieties (dimethoxynitrophenylethyl = DMNPE) at the phosphate 5’ and 3’-ends of both strands of siRNAs (Scheme 18) [74]. Here, again, the inhibition of gene silencing due to the caging moieties has not been complete, although much better than that with the backbone-modified siRNAs, in spite of the fact that the RNAi was fully restored after photoirradiation. One of the possible reasons for the partial inhibition of gene silencing by the photocaged siRNA (35% knockdown without photoirradiation) could be explained by the partial loss of terminal photoreactive units due to nuclease degradation. Friedman et al. have first improved their system using phosphorothioate (PS) internucleoside linkages to enhance nuclease resistance near the terminal caged phosphates preventing unwanted loss of the photoreactive moieties before photoirradiation [75]. This was the case when two PS linkages were introduced into each strand of caged siRNAs. Surprisingly, an increasing number of PS, up to 6 per strand, turned on the caged siRNA to an active species, probably because many PS linkages increased the affinity for DICER overcoming the blocking capacity of the caged ON. Finally, the best results were obtained when bulkier photolabile protecting groups (i.e., cyclododecyl-DMNPE = CD-DMNPE) were employed to cage siRNAs (Scheme 18) [76]. The system was efficient as the photocaged siRNA did not induce RNAi while it was fully deprotected under photolysis restoring the activity of the native siRNA.

**Scheme 19:**
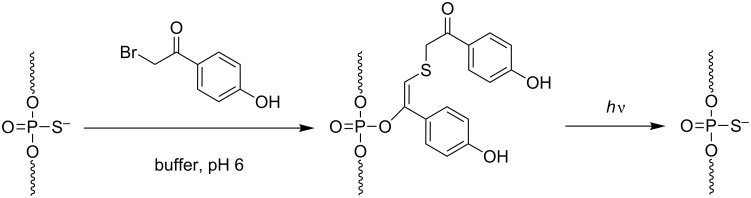
Post-synthetic introduction of a thioether-enol phosphodiester (TEEP) linkage into a DNAzyme by the selective reaction of a phosphorothioate linkage with 2-bromo-4’-hydroxyacetophenone followed by photodecaging, leading to a phosphodiester internucleoside linkage [77].

As stated previously, the introduction of a photoreactive moiety into the phosphodiester backbone of an ON with diazo compounds is not specific. Xiang et al. developed a more efficient and specific post-synthetic method. It is based on the reaction between a phosphorothioate derivative and 2-bromo-4’-hydroxyacetophenone to produce a phosphate protected with a thioether-enol phosphotriester, phenol substituted (TEEP, Scheme 19) [77]. The TEEP modification was introduced into “active sites” of 8–17 and 10–23 DNAzymes with good yields (>95%). The inhibition of the 8–17 DNAzyme activity by one modification was limited, whereas the photocaged ON with 3 modifications was totally inactive. Photoirradiation at 365 nm triggered the removal of the photoreactive moieties to phosphodiesters with up to 85% of activity recovery of the DNAzyme in vitro as in HeLa cells.

#### Modifications at the nucleobase

For selected reviews on this topic, see [79–80]. From all possible photoresponsive modifications introduced into ONs, modifications of the nucleobases are the most widely used for the regulation of gene expression under light activation. For this purpose various different approaches have been reported for the control of RNA translation (such as RNAi [81–83] and antisense [84–85], including splice switching of pre-mRNA [86] and DNAzymes [82,87]) and for the control of gene transcription (such as antigene strategy [88] and decoys [86,89] able to interact with transcription factors). Most of the photoresponsive units are introduced as protecting groups of nucleobases in the ONs. Consequently, the nucleobases cannot hybridize until photoirradiation. Another strategy much less studied than that where natural nucleobases are protected by photolabile groups is to use artificial photolabile nucleobases [90]. Generally, these modified nucleobases are introduced into ONs through their corresponding phosphoramidites.

**Scheme 20:**
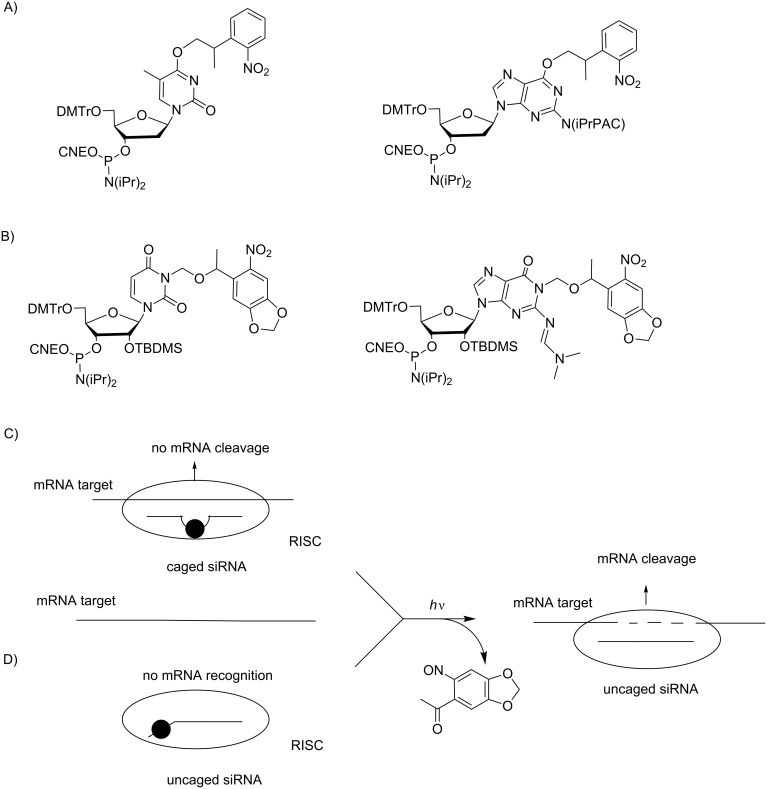
A) NPP dT and dG phosphoramidites [91–92] and B) NPOM U and G phosphoramidites [83] used to introduce photocaged nucleobases into siRNAs C) close to the argonaute cleavage site to prevent siRNA cleavage [81,83] and D) in the seed region to prevent mRNA recognition by the RISC complex [83].

**Photocaged approaches to inhibit translation:** Mikat and Heckel introduced deoxyguanosine and thymidine, respectively, protected at O6 and O4 with a 2-(2-nitrophenyl)propyl (NPP) group, into siRNA (Scheme 20A) [81]. The most efficient siRNAs targeting EFGP expression in transfected HeLa cells were those modified in the central part of the siRNA – that is, in the nucleobases neighboring the argonaute cleavage site of mRNA (Scheme 20C). These caged siRNAs were completely inactive until removal of the protecting groups with UV irradiation at 366 nm, whereas modifications surrounding the central part of the siRNA were less effective. It was argued that modified nucleotides in the central part of siRNA lead to a bulge of the siRNA–mRNA hybrid, disturbing the cleavage of mRNA by the RISC. Subsequently, Deiters used the same approach with photo 6-nitropiperonyloxymethyl (NPOM)-photocaged siRNAs synthesized from phosphoramidites of the caged uridine and guanosine ribonucleotides (Scheme 20B) [83]. As previously demonstrated, light activation of RNAi was confirmed in HeLa cells transfected with a GFP reporter gene but was also demonstrated with the silencing of the endogenous gene of the mitosis motor protein Eg5. In the same article, Deiters reported the study of siRNAs with caged nucleotides at the seed region of siRNA because the seed region is crucial for the recognition of mRNA target but does not affect the cleavage site (Scheme 20D). Two protected nucleotides in a siRNA totally prevented RNAi that is “turned on” after UV irradiation. Thus, the NPOM-protecting group induces reversible inactivation of siRNAs, demonstrating the importance of hybridization in the RNAi mechanism.

**Scheme 21:**
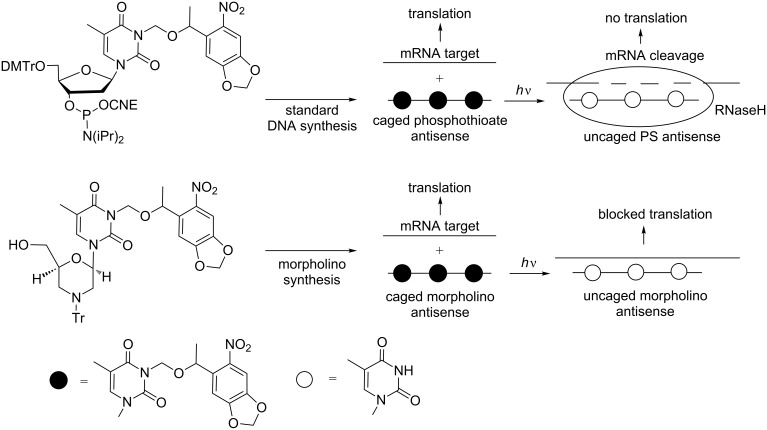
Introduction of the photocaged 3-NPOM nucleobase into phosphorothioate antisense and morpholino antisense to inhibit RNA translation though mRNA degradation by RNase H [84] or steric blocking [85].

Deiters et al. also applied the NPOM photosensitive group for gene silencing using antisense oligodeoxynucleotides (ODNs) in mouse fibroblast 3T3 cells transfected with the *Renilla* luciferase plasmid [84]. Three and four modifications partitioned along the sequence of the antisense ODN prevented hybridization to RNA targets and consequently inhibited the antisense activity blocking RNase H catalyzed degradation of mRNA. Upon irradiation at 365 nm, the NPOM groups were completely removed and the antisense activity was restored to the level of the uncaged ODN (Scheme 21). Photocaged NPOM thymine was further introduced into morpholino antisense ODNs [85] to block mRNA binding to the ribosome and, therefore, RNA translation. These morpholino ONs could inhibit the EGFP exogenous gene and chordin endogenous gene in zebrafish and Xenopus living embryos, only after UV photolysis at 365 nm (Scheme 21).

**Scheme 22:**
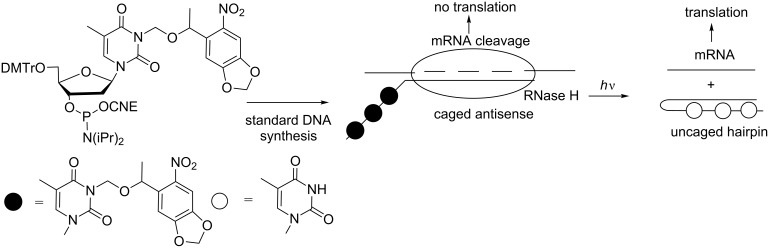
Control of the activity of an antisense ODN using a photocaged hairpin [82]. Formation of the hairpin suppresses hybridization of the antisense ODN with mRNA, which could be translated.

In the studies described above, photoirradiation “turns on” antisense activity, and ONs “turn off” gene translation. Photocaging can also be used to “turn off” antisense activity. For this purpose, the antisense ODN was linked to a complementary sequence (Scheme 22) [82]. The resulting hairpin could not associate with the mRNA. When the complementary sequence was photocaged with three NPOM thymidines, the hairpin was not formed, and the antisense hybridized with mRNA, preventing its subsequent translation by RNase H recruitment. Thus, photoirradiation causes hairpin formation and, therefore, “turns off” antisense activity.

**Scheme 23:**
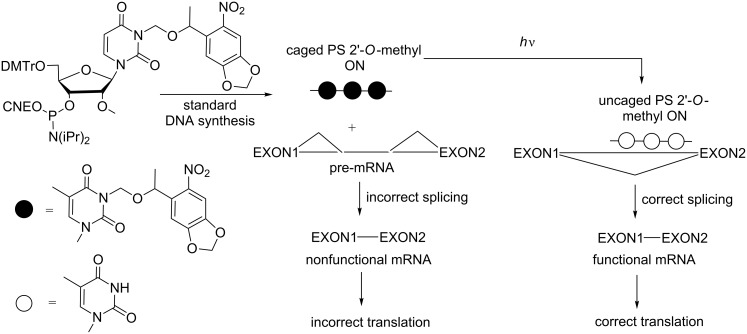
Control of alternative splicing using phosphorothioate (PS) 2’-OMe-photocaged ONs resulting from the incorporation of 3-NPOM 2’-OMe uridine phosphoramidite [86]. Photoirradiation activates the ODN, inducing a correct splicing.

Photocaged phosphorothioate (PS) ONs containing 2’-*O*-methyl nucleosides and two NPOM-protected 2’-OMe uridines in their sequences have also been used as splice-switching ONs (Scheme 23) [86]. The NPOM-protecting groups prevented ON hybridization with a β-globin intron aberrant splice site, inducing β-thalassemia in EFGP stably transfected HeLa cells, and the ON was not active until photoactivation.

**Scheme 24:**
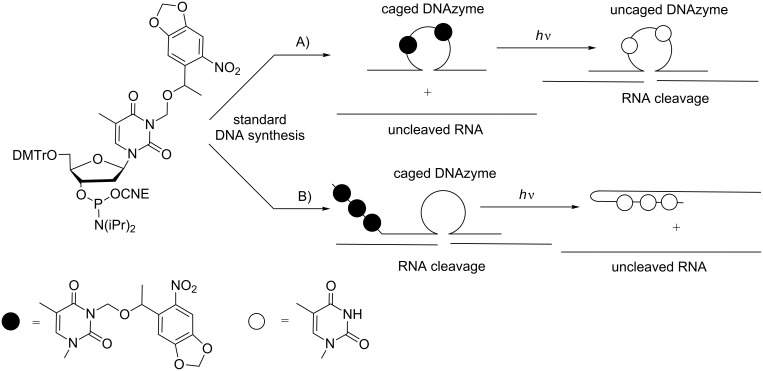
A) Light activation of a photocaged DNAzyme incorporating 3-NPOM thymidine in its catalytic site [87]; B) light deactivation of a photocaged DNAzyme by formation of an inactive hairpin [82].

In 2007, Deiters et al. described the recovery under UV irradiation of the catalytic activity of a DNAzyme possessing in its catalytic loop a thymidine caged with the NPOM-protecting group in N3 of thymine (Scheme 24A) [87]. In this approach, the DNAzyme was light activated. Some years after, the same group showed a light deactivation process using a caged hairpin (Scheme 24B) [82]. In this case, the catalytic site of DNAzyme was not caged, but it was associated or linked to a complementary photocaged ON, and the DNAzyme could induce cleavage of a mRNA target. Once deprotected under UV light, this complementary ON hybridized to the catalytic site and inhibited the effect of DNAzyme, allowing mRNA translation.

**Scheme 25:**
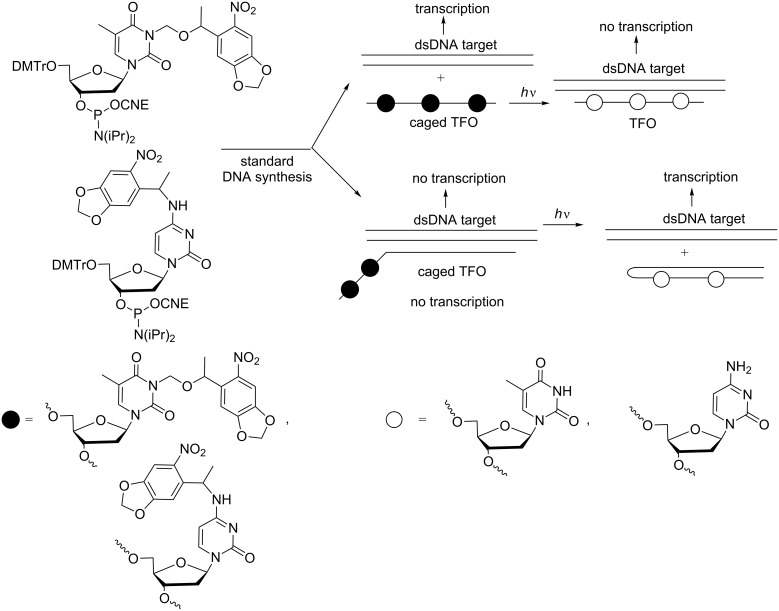
Incorporation of 3-(6-nitropiperonyloxymethyl) (NPOM) thymidine and 4-nitropiperonylethyl (NPE) deoxycytidine phosphoramidites into TFOs and light inhibition and light activation of gene transcription using caged TFOs and caged hairpin TFOs, respectively [88].

**Photocaged approaches to inhibit transcription:** Similarly to antisense and DNAzymes, two similar photocaged approaches have been explored to activate or deactivate triplex-forming ONs (TFOs). These approaches inhibit or elicit gene transcription, respectively [88]. Photocaging of TFOs using NPOM-protected nucleobases prevented the formation of a triple helix with a dsDNA target, consequently permitting gene transcription (Scheme 25). Inversely, when photoirradiation removes the protecting groups, the ON creates a triple helix, hindering gene transcription. By contrast, when the TFO was linked to a caged complementary sequence, the construct could block transcription until photoirradiation led to the formation of the hairpin unable to interact with dsDNA. These photocaged DNAzymes were tested as gene silencing agents to target the reporter gene DsRed in eukaryotic cells.

**Scheme 26:**
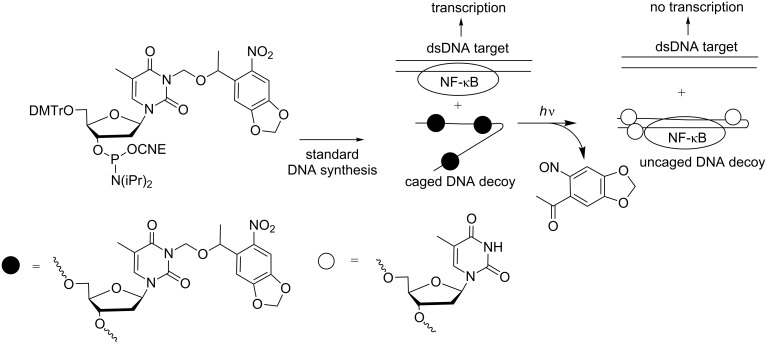
Synthesis of a photocaged DNA decoy from a 3-NPOM thymidine phosphoramidite and release of the NPOM protecting group under photolysis, allowing the decoy to organize into a hairpin that can bind to the NF-κB transcription factor [89].

The first illustration of a photocaged DNA decoy used for the photocontrol of gene expression in mammalian cells was reported in 2011 by Deiters et al [89]. As generally observed, the protecting groups of the nucleobases disturb base pairing that the hairpin decoy could not be formed. The decoy is thus inactive, and the NF-κB transcription factor binds to the NF-κB binding site of an alkaline phosphatase gene to allow transcription. Photodecaging permits hairpin formation, and the active decoy can then bind to NF-κB and compete with the NF-κB binding site of the gene, leading to the inhibition of gene transcription (Scheme 26).

**Scheme 27:**
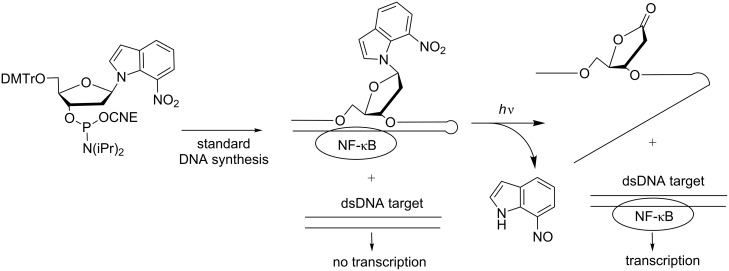
Synthesis of a caged DNA decoy hairpin containing a 7-nitroindole nucleotide and release of the modified nucleobase under photolysis, leading to an abasic lactone-containing ON that cannot form a hairpin and associate with NF-κB [90].

It is noteworthy that the photodeactivation of DNA decoys was also described using a modified photocleavable nucleobase [90]. 7-Nitroindole nucleotides incorporated in a DNA decoy did not suppress hairpin formation so that NF-κB could bind to the decoy (Scheme 27). Under UV irradiation, the nucleobase was photolyzed, releasing an abasic lactone and lowering the affinity for NF-κB targets. This approach is attractive to “turn on” the transcription upon UV light. However, until now, the effect on gene transcription was not reported.

#### Modifications at the sugar 2’-OH

**Figure 5:**
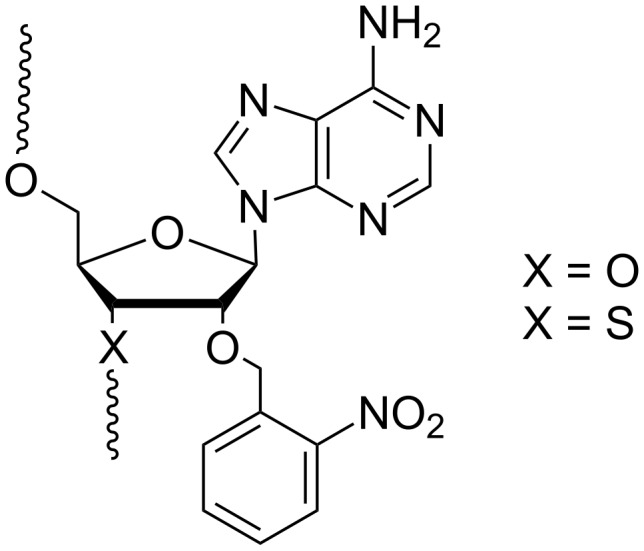
Caged-2’-adenosines used by MacMillan et al [93–94] (X = O) and Piccirilli et al [95] (X = S) to study RNA mechanisms.

Light-dependent regulation of gene expression resulting from the interaction of 2’-O-photocaged ONs with the genetic material is not documented compared with ONs modified at phosphates or nucleobases [68]. Generally, what is sought is to suppress the chemical reactivity of this nucleophilic hydroxy function involved in a transesterification reaction that modifies the RNA substrate of the ribozyme but not the catalytic ON itself (Figure 5) [93–94]. This method is inappropriate for potential therapeutic applications. Curiously, to our knowledge, these modifications have not been exploited for the regulation of RNA interference.

**Use of photolabile linkers:** For a selected review, see [96]. In this approach, the photolabile moieties are not nucleotide protecting groups within ON but are non-nucleoside moieties linking different ONs or both ends of the same ON together or ONs to other molecules. Most frequently, except for circular ONs, photoirradiation cuts the construct into small fragments that induce a change in the biological activity. Photolysis of circular ONs provides linear full-length ONs. Compared to caged nucleobases that directly interact with their nucleic acid targets, photosensitive linkers do not interact but can organize the ONs into specific structures capable of or not interfering with their nucleic targets.

Control of gene expression with photocaged linker-modified ONs has been mostly used for light activation or deactivation of antisense inhibition of RNA translation by Tang and Dmochowski [97–102]. Nevertheless, they were also used to regulate the catalytic effect of DNAzymes [103] and to control alternative splicing as reported by Deiters et al [86].

**Scheme 28:**
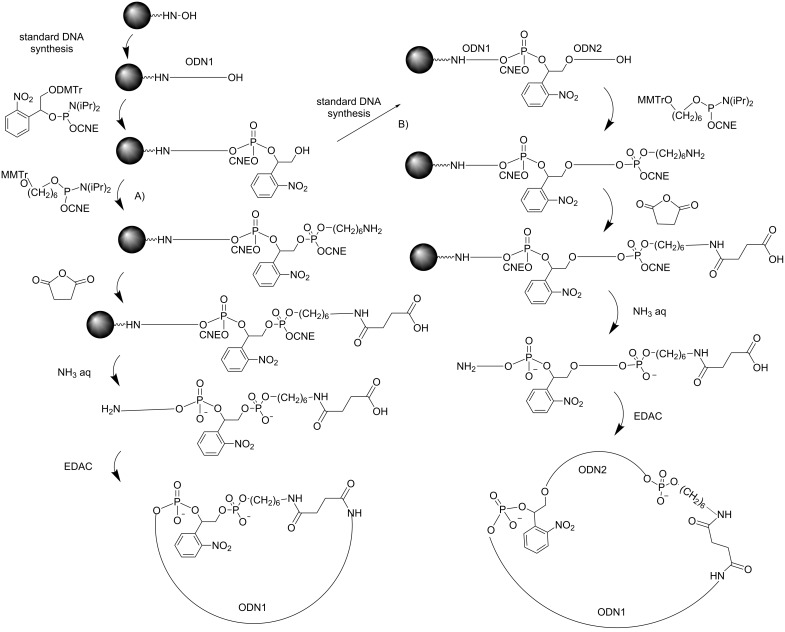
Synthesis of circular ODNs containing a photolabile linker as described by Tang et al. [101,104].

Two chemical approaches exist to introduce a photoresponsive linker. The first is a post-DNA synthesis process using a heterobifunctional moiety that connects two ONs bearing complementary functionalities. The conjugation of two amino and thiol-terminated ONs with a photoresponsive 2-nitrophenylethanol unit bearing a *N*-hydroxysuccinimide ester and maleimide is an example [97,100]. In the second approach, the linker is incorporated as a phosphoramidite derivative bearing a protected hydroxy function for ON elongation using standard solid-support DNA synthesis [86,103]. This approach is beneficial because several photoactivatable phosphoramidites are commercially available. Beside these two strategies, miscellaneous processes were employed for the synthesis of circular DNA. Dmochowski used the phosphoramidite ligation method between two ONs, and then, the construct was phosphorylated at its 5’-end. After deprotection, the circularization was performed using a single-strand DNA ligase [103]. In 2010, Tang introduced a photoresponsive 1-(2-nitrophenyl)-1,2-ethanediol phosphoramidite at the end of a solid-supported 3’-amino ON (Scheme 28) [101]. This step was followed by the incorporation of an amino-C6-linker phosphoramidite. Before cleavage from the solid support, the 5’-amino functionality was reacted with succinic anhydride, yielding an ON with an amino group at the 3’ end and a carboxyl group at the 5’ end after deprotection and cleavage from the support (Scheme 28A). Both ends were then chemically linked using water-soluble 1-ethyl-3-(3-dimethylaminopropyl)carbodiimide, hydrochloride (EDAC, synthetic yield 20–40%). More recently, the same author followed a quite different approach (Scheme 28B) [104]. After incorporation of the photoresponsive phosphoramidite unit into a 3’-amino solid-supported ON, elongation was ongoing, and then, the aminolinker phosphoramidite was incorporated at the 5’-extremity. The reaction with succinic anhydride followed by the deprotection produced a 5’-carboxyl 3’-amino ON. Both ends, as previously described, were then connected using EDAC with isolated yields of 30–40%.

**Scheme 29:**
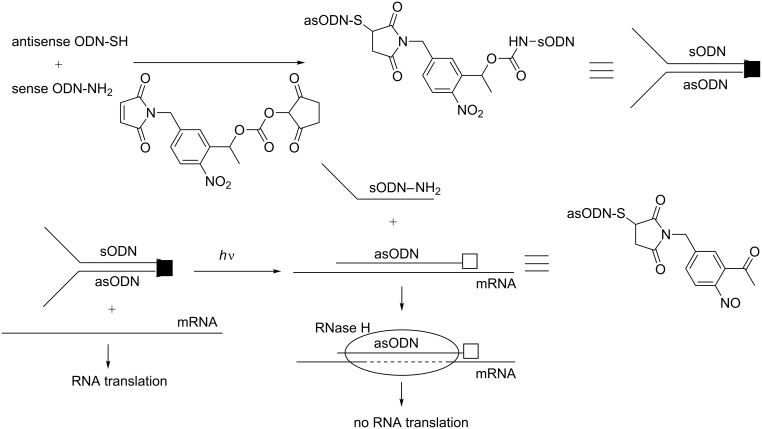
Control of RNA digestion with RNase H using light activation of a photocaged hairpin [97].

**Photocleavable linkers in antisense ONs:** Tang and Dmochowski have introduced the 2-nitrophenylethyl-containing linker in the loop of a DNA hairpin where an antisense DNA strand (20-mer) was linked to a shorter complementary ODN (Scheme 29, 12 base pairs). This hairpin was very stable, and the antisense ODN did not hybridize to its RNA target and could not elicit RNA degradation by RNase H. Upon UV irradiation, cleavage of the linker in the hairpin occurred, and the resulting duplex became much less stable, permitting the antisense ODN to hybridize to RNA and turn on its antisense activity [97]. Therefore, while the hairpin induced only 5% degradation of the 15-mer RNA after 1 hour, 66% of RNA degradation was observed upon UV irradiation.

The same authors applied their concept of antisense photocaged DNA hairpins to the inhibition of dC-myb expression in human leukemia cells [105]. The concept was further extended to PNA [99] and morpholino antisense ONs in zebrafish embryos [98] to block physical RNA translation by interaction with the ribosome.

**Scheme 30:**
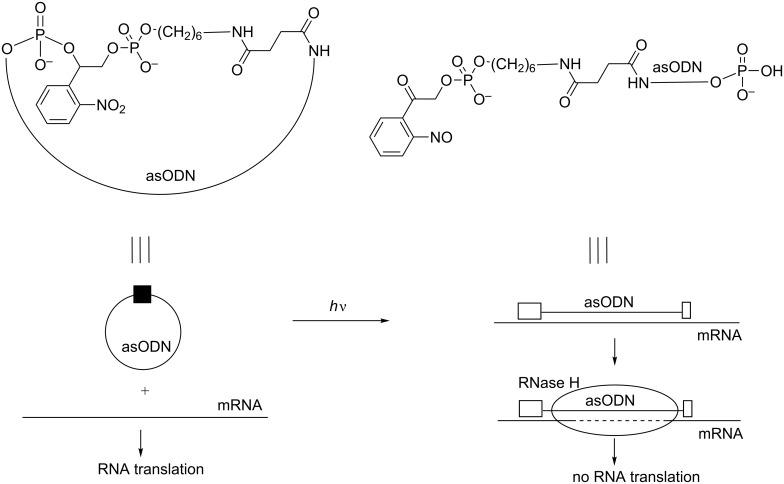
Photocontrol of RNA degradation using caged circular antisense ODNs containing a photoresponsive linker [101].

Another method to cage an antisense ODN is to circularize it (Scheme 30) [101]. For this purpose, a single photocleavable linker connected both ends of the ONs as described above. The circular ONs have different lengths, and some of them have a “hairpin-like” or a “dumbbell-like” structure. The circularization of longer ONs (30–40-mers) partially prevented their hybridization to a 40-mer RNA so that RNase H degradation of the RNA target was observed. In this case, photoirradiation at 350 nm activated a 2 to 3-fold increase in RNA degradation by RNase H. A shorter circular ON produced better results because the photocaged ON did not elicit target degradation by RNase H, while photoactivation turned on the antisense activity with a 20-fold increase. The use of circular ONs was further extended by the same author to a steric block GFP RNA translation in transfected HeLa cells by 2’-*O*-methyl phosphorothioate circular ONs [104] and to morpholino-caged ONs in zebrafish embryos to effectively control δ-catenin-2 and no tail gene expression [102].

**Scheme 31:**
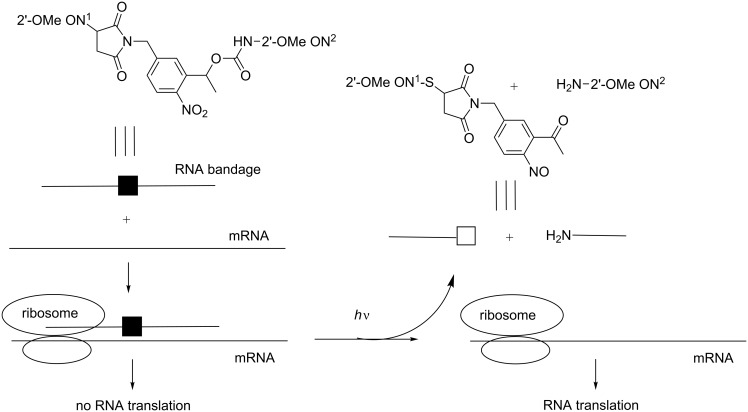
Control of RNA translation using an “RNA bandage” consisting of two short 2’-OMe ONs linked together with a photosensitive linker [100].

In the reports cited above, the photocaged ONs are light activated. In the subsequent studies, the photocaged ONs are deactivated by light. As a first example, Dmochowski et al. described the use of two 6 to 12-mer 2’-OMe RNAs linked together through a photocleavable linker and a 4-base gap in 2008 (Scheme 31). These “RNA bandages” hybridized to an RNA target and blocked its translation. Photoirradiation caused the release of linked short entities that were consequently unable to interact efficiently with the RNA target and obviously blocked its translation [100]. The ability of light to turn off the antisense activity of these “RNA bandages” and to promote gene expression of a GFP transcript was evaluated in rabbit reticulocyte lysates. The most effective photoregulation was obtained using an asymmetric bandage with a short 5’ 2’-OMe RNA and a low melting temperature near the start codon linked to a second longer 2’-OMe RNA through the photolabile linker.

**Scheme 32:**
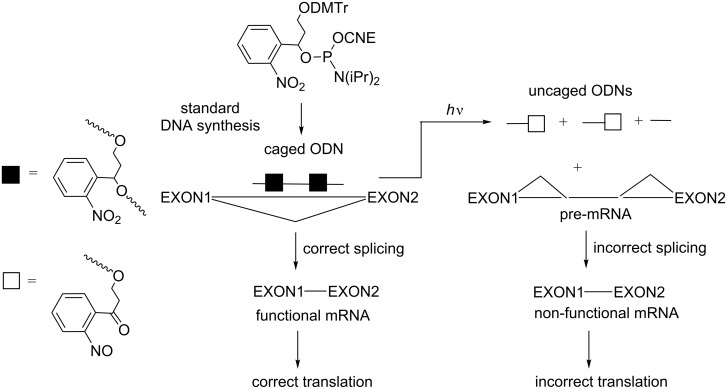
Control of alternative splicing using photocaged ONs resulting from the incorporation of an *o*-nitrobenzyl responsive moiety as its phosphoramidite [86]. Photoirradiation deactivates the ODN, inducing incorrect splicing.

Another study relating to light deactivation of a caged ON was reported by Deiters et al., who introduced two photoresponsive *o*-nitrobenzyl linkers into splice-switching ONs (Scheme 32). The use of antisense ONs to correctly aberrant expression during pre-mRNA splicing showed great potential to correct resulting diseases. The photocaged antisense ONs interacted with pre-mRNA and blocked aberrant intron sequences, permitting correct exon splicing and thus correct gene EGFP expression in transfected HeLa cells [86]. Upon UV irradiation, the caged ON fragmented into three shorter pieces, which did not hybridize to pre-mRNA so that the gene was not expressed (on→off effect).

**Scheme 33:**
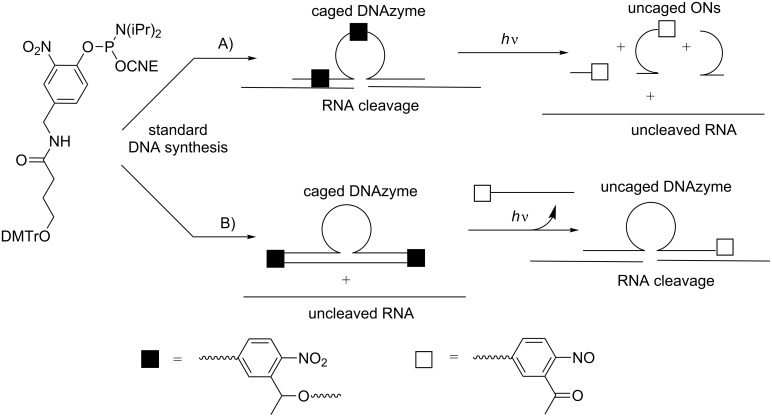
A) Light deactivation of a photocaged DNAzyme incorporating one photocleavable spacer in its catalytic site and another in the recognition site; B) light activation of a circular photocaged DNAzyme formed through the hybridization and ligation of the DNAzyme with a complementary strand [103].

**Photocleavable linkers in DNAzymes:** Dmochowski et al. have demonstrated that the replacement of thymidine dT8 in the 10–23 DNAzyme with a photocleavable linker introduced as its phosphoramidite in the DNA sequence did not suppress the catalytic effect of the DNAzyme [103]. Unexpectedly, two smaller ONs resulting from cleavage of the linker through photoirradiation also showed a catalytic effect although a reduced one (Scheme 33A). The best difference between the caged DNAzyme and the resulting decaged products was obtained with DNAzyme incorporating two modifications: one in the catalytic site and the other in the recognition site of the DNAzyme. It was argued that in this case, the photolysis produced three ONs, which were too small to hybridize to RNA, and induced its cleavage (on→off effect).

Another approach described in the same article involved a circular DNAzyme incorporating an ON-blocking strand complementary to the recognition site of DNAzyme and joint to the DNAzyme through two linkers at its 5’- and 3’-ends (Scheme 33B). Thus, the DNAzyme was inefficient to hybridize to RNA and, consequently, could not induce its cleavage. Photoirradiation released the free DNAzyme, which then induced the catalytic cleavage of RNA (off→on effect).

**Scheme 34:**
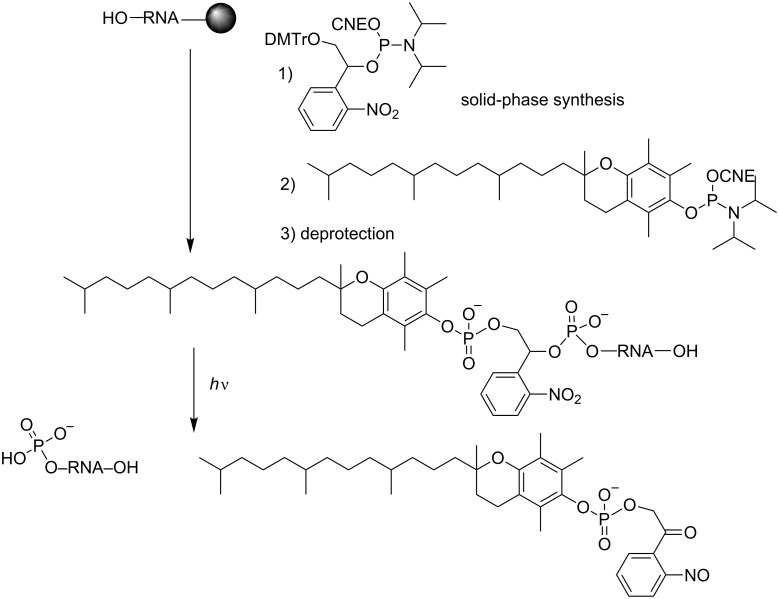
Solid-phase synthesis of a caged vit E-siRNA conjugate and its release upon UV irradiation [106].

**Photocleavable linkers in siRNA conjugates:** Tang et al. have described the control of RNAi in HEK293 cells using photocaged siRNAs conjugated with a 5’-terminal vitamin E (vit E) through a photolabile linker and a 4-base gap [106]. Both, the linker and vit E were introduced into siRNAs using their corresponding phosphoramidites (Scheme 34). In this concept, the photoresponsive unit did not directly interfere with the biological activity of the photocaged conjugate. However, vit E, which interacted with the binding protein targets, prevented the association of ON with the RNAi machinery. The photolysis released ON from the vitamin, and siRNA activity was activated.

### Chemical-responsive ONs

**Scheme 35:**
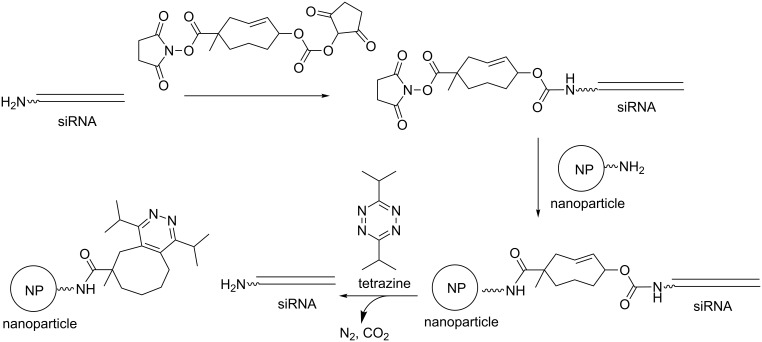
Synthesis of a siRNA conjugated to a nanoparticle (NP) via a cyclooctene heterolinker from a siRNA-NH_2_ and an NP-NH_2_ [107]. The conjugate does not induce gene silencing until tetrazine triggers siRNA release.

Light or heat is an external physical regulatory element compared to glutathione, for example, which is an internal chemical regulatory element, or carboxyesterases and reductases, which are internal biochemical regulatory stimuli. The use of an external chemical factor to trigger the activity of ON prodrugs has been rarely reported in the literature. Recently, however, Royzen reported such an approach to control in-cell siRNA activity [107]. To this end, 3’-amino siRNA was linked to amino-functionalized nanoparticles (NP) through a bifunctional trans-cyclooctene heterolinker (Scheme 35). These conjugates cannot interfere with RISC and do not allow gene silencing until tetrazine releases the ON from the nanoparticle by an inverse-electron demand Diels–Alder reaction with biocompatible tetrazine. The gene silencing of exogenous GFP and endogenous CDK8 genes in MDA-MB-231 breast cancer cells was demonstrated.

## Conclusion

The interest in stimuli-responsive ONs to control gene expression has increased in recent years. This prodrug approach, as most of the permanent ON modifications, aims to overcome the limitations of ONs due to their poor extracellular and intracellular stability, low efficiency of intracellular delivery to target cells or tissues and possible off-target gene silencing, immunostimulation and other side effects. However, for stimuli-responsive ONs, the desired effect is that of "natural" ONs obtained after transformation in response to a stimulus that may be internal or external, biochemical, chemical or physical. Compared to permanent modifications, transient modifications have the great advantage to regulate the activity of ONs as a function of stimuli acting as switches.

Most of the examples of stimuli applicable to ON prodrugs have been gathered in this review. Physical stimuli such as heat and light can be easily controlled by the operator, whereas biochemical stimuli such as enzymes act on a difference between the contents of the intracellular and the extracellular compartment. Creative and ingenious chemistry was used to design all these stimuli-responsive modifications, most of which have been evaluated at least in vitro and some of which seemed promising. Nevertheless, among the stimuli-responsive ONs described in this review, most of them have been tested in cellulo on reporter gene models except for a few studies on specific genes in embryos for some photocaged ONs [85,102]. In addition, it is noteworthy that for the first time, a biological effect was measured in mice with siRNA prodrugs containing charge-neutralizing phosphotriester linkages [43] and these data are promising for ON prodrug-based approaches. The numerous literature references on light-responsive ONs compared to other stimuli-responsive ONs deserve to be highlighted to show how much effort was put on this subject during this last decade. Indeed, this may be explained by the fact that photoirradiation is the major and the simplest method to control the response of caged ONs both, in time and in space.

We hope this review provides insight into the available transient modifications to make efficient ON prodrugs. To date, the successful approach to obtain ON therapeutics based on a prodrug strategy remains unresolved, but the recent report on an example of a chemical external stimulus opens an exciting future in the prodrug field [107]. The abbreviations used in this review are listed in Table 1.

**Table 1:** List of abbreviations.

abbreviation	full length

A	adenine
Ac	acetyl
AMPrOM	2-amino-2-methylpropionyloxymethyl
AMEBuOM	2-aminomethyl-2-ethyl-butyryloxymethyl
AON	antisense oligonucleotide
Boc	*tert*-butoxycarbonyl
Bn	benzyl
BuNH_2_	butylamine
C	cytosine
CNE	cyanoethyl
DBU	1,8-diazabicyclo[5.4.0]undec-7-ene
DMTr	dimethoxytrityl
DMTSF	dimethyl(methylthio)sulfonium tetrafluoroborate
dT	thymidine
DMNPE	dimethoxynitrophenylethyl
CD-DMNPE	cyclododecyl-DMNPE
EDAC	1-ethyl-3-(3-dimethylaminopropyl) carbodiimide
Et	ethyl
*fma*	2-(*N*-formyl-*N*-methyl) aminoethyl
Fmoc	fluorenylmethoxycarbonyl
G	guanine
GFP	green fluorescent protein
GMEBuOM	2-guanidinomethyl-2-ethyl-butyryloxymethyl
GSH	glutathione
HIV	human immunodeficiency virus
iPr	isopropyl
iPrPac	isopropylphenoxyacetyl
Lev	levulinyl
Me	methyl
MDTM	methyldithiomethyl
miRNA	micro ribonucleic acid
MMTr	monomethoxytrityl
MOE	methoxyethyl
NADH	nicotinamide adenine dinucleotide
NB	nitrobenzyl
NP	nanoparticule
NPE	4-nitropiperonylethyl
NPOM	6-nitropiperonyloxymethyl
NPP	2-(2-nitrophenyl)propyl
ON	oligonucleotide
ODN	oligodeoxyribonucleotide
Pac	phenoxyacetyl
PiBuOM	phenylisobutyryloxymethyl
PivOM	pivaloyloxymethyl
PNA	peptide nucleic acid
PrOM	propionyloxymethyl
PS	phosphorothioate
psc	phenylsulfonylcarbamoyl
Q-linker	hydroquinone-*O,O’*-diacetic acid
RNAi	RNA interference
RNN	ribonucleic neutral
RSSM	alkyldithiomethyl
A-SATE	aldehyde SATE
Me-SATE	S-acetylthioethyl
*t-*Bu-SATE	*S*-pivaloylthioethyl
siRNA	small interfering ribonucleic acid
T	thymine
TAR	trans-activation response
TAT	transactivator of transcription
TBA	thrombin-binding DNA aptamer
TBDMS	*tert*-butyldimethylsilyl
Tc-DNA	tricyclo-DNA
tc^ee^-T	ethyl tricyclo-thymine
tc^hd^-T	hexadecyl tricyclo-thymine
TEEP	thioether-enol phosphodiester
TPP	triphenylphosphonium
U	uracil
vit E	vitamin E
